# On the road to universal health care in Indonesia, 1990–2016: a systematic analysis for the Global Burden of Disease Study 2016

**DOI:** 10.1016/S0140-6736(18)30595-6

**Published:** 2018-08-18

**Authors:** Nafsiah Mboi, Indra Murty Surbakti, Indang Trihandini, Iqbal Elyazar, Karen Houston Smith, Pungkas Bahjuri Ali, Soewarta Kosen, Kristin Flemons, Sarah E Ray, Jackie Cao, Scott D Glenn, Molly K Miller-Petrie, Meghan D Mooney, Jeffrey L Ried, Dina Nur Anggraini Ningrum, Fachmi Idris, Kemal N Siregar, Pandu Harimurti, Robert S Bernstein, Tikki Pangestu, Yuwono Sidharta, Mohsen Naghavi, Christopher J L Murray, Simon I Hay

**Affiliations:** a Centre for Strategic and International Studies, Jakarta, Indonesia; b National Commission for Tobacco Control, Jakarta, Indonesia; c Independent consultant, Jakarta, Indonesia; d Central Bureau of Statistics, Jakarta, Indonesia; e Faculty of Public Health, University of Indonesia, Depok, Indonesia; f Eijkman-Oxford Clinical Research Unit, Jakarta, Indonesia; g National Development Planning Agency (BAPPENAS), Jakarta, Indonesia; h Department of Anthropology, McGill University, Montreal, QC, Canada; i Institute for Health Metrics and Evaluation, University of Washington, Seattle, WA, USA; j Department of Public Health, Universitas Negeri Semarang, Semarang City, Indonesia; k Graduate Institute of Biomedical Informatics, College of Medical Science and Technology, Taipei City, Taiwan; l Sriwijaya University, Palembang, Indonesia; m Social Security Administering Body for Health, Jakarta, Indonesia; n World Bank, Jakarta, Indonesia; o Department of Global Health, Rollins School of Public Health, Emory University, Atlanta, GA, USA; p Department of Global Health, College of Public Health, University of South Florida, Tampa, FL, USA; q Lee Kuan Yew School of Public Policy, National University of Singapore, Singapore; r Field Epidemiology Training Program Indonesia, Jakarta, Indonesia; s Oxford Big Data Institute, Li Ka Shing Centre for Health Information and Discovery, University of Oxford, Oxford, UK

## Abstract

**Background:**

As Indonesia moves to provide health coverage for all citizens, understanding patterns of morbidity and mortality is important to allocate resources and address inequality. The Global Burden of Disease 2016 study (GBD 2016) estimates sources of early death and disability, which can inform policies to improve health care.

**Methods:**

We used GBD 2016 results for cause-specific deaths, years of life lost, years lived with disability, disability-adjusted life-years (DALYs), life expectancy at birth, healthy life expectancy, and risk factors for 333 causes in Indonesia and in seven comparator countries. Estimates were produced by location, year, age, and sex using methods outlined in GBD 2016. Using the Socio-demographic Index, we generated expected values for each metric and compared these against observed results.

**Findings:**

In Indonesia between 1990 and 2016, life expectancy increased by 8·0 years (95% uncertainty interval [UI] 7·3–8·8) to 71·7 years (71·0–72·3): the increase was 7·4 years (6·4–8·6) for males and 8·7 years (7·8–9·5) for females. Total DALYs due to communicable, maternal, neonatal, and nutritional causes decreased by 58·6% (95% UI 55·6–61·6), from 43·8 million (95% UI 41·4–46·5) to 18·1 million (16·8–19·6), whereas total DALYs from non-communicable diseases rose. DALYs due to injuries decreased, both in crude rates and in age-standardised rates. The three leading causes of DALYs in 2016 were ischaemic heart disease, cerebrovascular disease, and diabetes. Dietary risks were a leading contributor to the DALY burden, accounting for 13·6% (11·8–15·4) of DALYs in 2016.

**Interpretation:**

Over the past 27 years, health across many indicators has improved in Indonesia. Improvements are partly offset by rising deaths and a growing burden of non-communicable diseases. To maintain and increase health gains, further work is needed to identify successful interventions and improve health equity.

**Funding:**

The Bill & Melinda Gates Foundation.

## Introduction

The Republic of Indonesia (henceforth referred to as Indonesia), the world's largest archipelago, consists of more than 17 000 islands straddling the equator, stretching 5200 km from east to west. Indonesia is the fourth most populous country and the largest Muslim-majority nation in the world, with some 260 million inhabitants. More than half of all Indonesians live on Java, with the rest of the population distributed unevenly across the archipelago, presenting substantial challenges for governance, communication, transportation, and the equitable availability of basic health services.

To help to address these challenges, in 1999 the Indonesian Government passed law 22, initiating the process of decentralisation, with further legislation passed in 2004. In the devolved system, city and district heads (the mayor and regent, or *bupati*) were given primary responsibility for health care, with technical and financial support from the national or provincial level as needed. This transition has meant greater autonomy for local and regional heads of government to serve their diverse populations.

Total health expenditure in Indonesia per person has tripled since 2000, with private expenditure accounting for two thirds of all health spending, primarily for out-of-pocket expenses.^1^ In 2004, growing concerns with productivity, equity, and the security of human capital led to the national adoption of a comprehensive social security system.^2^ Health coverage was scaled up almost immediately, with a limited programme for the poor initiated in 2005 and expanded in 2008, and the addition of coverage for antenatal and postnatal care and delivery services for pregnant women in 2010.

At the start of 2014, the Indonesian Government launched its National Social Health Insurance Scheme (known as the *Jaminan Kesehatan Nasional*, or JKN), which aims to provide health coverage for all Indonesians, with the government paying the modest premiums of the poor and near poor.^3^ For the population whose premium is covered by the government, enrolment in the programme has increased from 86·4 million people in 2014 to 111·6 million in November, 2017 (with 92·2 million people funded at the national level and 19·4 million at the local level).^4^ The government has set 2019 as its target for the enrolment of 95% of the population—the functional achievement of universal health care.^5^

**Research in context**

**Evidence before this study**

Routinely collected health statistics from Indonesia have informed previous Global Burden of Disease (GBD) studies, which, in 2016, estimated health loss for 333 causes of death and disability and 84 risk factors in 195 locations from 1990 to 2016. Since 1990, Indonesia has completed many nationally representative surveys and censuses; the GBD studies include these and other data subjected to rigorous inclusion criteria in the estimation process. Yet, outside of the GBD studies, health data in Indonesia are infrequently aggregated in a systematic manner, and the full range of diseases, injuries, and risk factors is not consistently captured. Because of the country's size, diversity, and widespread socioeconomic disparities, the comprehensive analytic framework of the GBD studies can yield improved population health measurement.

**Added value of this study**

This analysis represents the largest systematic effort to date to quantify levels and long-term trends in mortality, disability, and risk-attributable burden of diseases and injuries in Indonesia. The GBD 2016 study allows for comprehensive comparisons across time and between comparator countries, providing new insights into health trends and identifying areas meriting heightened policy attention. GBD 2016 made substantial methodological improvements from GBD 2015, and included estimates for an additional 21 causes of death and disability. Our study comes at a crucial time for benchmarking and priority setting for health spending and programme development in Indonesia, as the country moves towards implementing universal health care by 2019.

**Implications of all the available evidence**

Health in Indonesia has substantially improved since 1990. However, these improvements have not been homogeneous and a double burden of communicable and non-communicable disease is placing increasing strain on the nation's health system and consequently its ambitious efforts to achieve universal health care. Data reflecting regional differences are needed to target improvements to specific areas that are lagging behind or have distinctive challenges. In tandem with more integrated models of service delivery, an emphasis on the early detection and prevention of non-communicable diseases and risk factors might help to alter the course of Indonesia's growing epidemic of non-communicable diseases and related disability. In particular, high levels of tobacco smoking threaten to undermine progress and increase already high rates of tobacco-related death and disability.

By July 1, 2017, JKN had contracts with 26 000 health facilities and providers across Indonesia's 34 provinces, serving 180·7 million total members—68% of Indonesia's total population. More than 57 trillion Indonesian Rupiah (US$4·8 billion) have been spent to serve 100 million outpatient episodes and 40 million inpatients.^6^ The JKN emphasises primary care and improved procurement, distribution, and utilisation of key medicines. Its main objectives include increasing equity in access to health care, improving health outcomes, and keeping health-care costs down.^7^

Efforts to achieve universal health care, particularly in poorer and isolated areas, will require effective health promotion and the extension of quality health care to all Indonesians.^1^, ^2^, ^8^, ^9^ Given the diverse range of geography and economic status across the country, regular comprehensive assessment of disparities in morbidity, mortality, and disability patterns and their causes is needed. In this Article, we use the results of the Global Burden of Diseases, Injuries, and Risk Factors (GBD) Study 2016 to examine Indonesia's health transition from 1990 to 2016 against comparator nations. The GBD study makes domestic and international comparison possible and provides valuable additional data and perspective in examining the nation's health status. The results of our study will help to identify gaps and develop national-level responses that can support subnational leaders and managers to improve the availability, accessibility, appropriateness, quality, and equity of health care.

This Article will serve as an important reference point for a subnational study anticipated later in 2018. The analysis will also form part of an increasing body of work of the GBD collaborator teams with a strong local interest in producing results and evidence that directly serve policy needs.^10^, ^11^, ^12^, ^13^, ^14^ Collaborators have further produced a series of subnational estimates in 12 countries, allowing local policy makers to set priorities and track changes at finer administrative levels.^15^, ^16^, ^17^, ^18^ Here, we present the first comprehensive national-level assessment of the burden of disease in Indonesia to delineate prominent health challenges on the nation's journey to universal health care.

## Methods

### Overview

For this analysis, Indonesia-specific estimates of life expectancy at birth, healthy life expectancy (HALE), cause-specific mortality, years of life lost (YLLs), years of life lived with disability (YLDs), disability-adjusted life-years (DALYs), and related risk factors are reported along with national estimates from seven comparator countries to facilitate comparison and benchmarking between 1990 and 2016. Brazil, India, Malaysia, the Philippines, Thailand, Turkey, and Vietnam were chosen as comparator countries on the basis of their geographical proximity, similar sociodemographic levels, or the presence of Muslim majority in the population.

This study complies with the Guidelines for Accurate and Transparent Health Estimates Reporting, shown in the appendix (pp 2–6).^18^ Additional information on all data sources used in GBD 2016 can be found on the Global Health Data Exchange website. GBD 2016 results for all years and locations can be explored further with dynamic data visualisations.

### Mortality

Cause-specific mortality was estimated for each age, sex, location, and year. Data were evaluated for completeness and misclassification, and cleaned, disaggregated, and mapped to International Classification of Diseases (ICD) codes. Deaths with non-specific or impossible codes, termed garbage codes, were redistributed to appropriate ICD codes by level in the GBD hierarchy prior to modelling. A complete list of data used to generate mortality estimates for Indonesia can be found in the appendix (pp 7–15). The most commonly used estimation method for cause-specific mortality was cause of death ensemble modelling using the GBD cause of death database.^19^ This process is explained in more detail in the appendix (p 16). YLLs were computed by multiplying the number of deaths in each age group by a reference life expectancy from analyses of all-cause mortality.

### Morbidity

For each combination of age, sex, year, and location, most prevalence and incidence estimates were generated using Bayesian meta-regression methods (DisMod-MR 2.1, as described in the appendix, p 16), with cause-specific exceptions outlined in other publications.^20^, ^21^ YLDs were subsequently calculated by taking into account disease severity, exclusivity, and comorbidity; further details on the calculation can be found in the appendix (p 16).^21^

### Life expectancy, HALE, and DALYs

The calculation of standard life expectancy at birth and for specific age groups was done using the world population age standard.^22^ HALE at birth and in specific age groups was calculated using YLD estimates and GBD life tables on the basis of methods originally developed by Sullivan.^23^, ^24^ DALYs were calculated by summing YLLs and YLDs.

### Risk factor estimation

Minimum risk level, exposure, risk, and attributable burden due to 84 behavioural, environmental and occupational, and metabolic risks or clusters of risks were estimated using the GBD comparative risk assessment framework.^25^ The attributable burden for each risk-outcome pair was calculated as the total deaths or YLLs multiplied by the population attributable fraction.

### Uncertainty levels

Uncertainty levels were propagated at multiple stages throughout the GBD modelling process.^26^ Uncertainty for mortality and YLLs reflected uncertainty in the levels of all-cause mortality and in the estimation of each mortality cause, in each age group, sex, and year. Uncertainty in the disability weight for each sequela was propagated into the estimates of YLDs for each disease and injury. A sample of 1000 draws was taken from the posterior distribution of each estimation step; aggregation of uncertainty across age, sex, and location was done on each draw, assuming independence of uncertainty. The lower and upper uncertainty intervals (UIs) represent the ordinal 25th and 975th draws of each quantity and attempt to describe modelling as well as sampling error.

### Socio-demographic Index and expected mortality analysis

The Socio-demographic Index (SDI) is a summary measure that takes total fertility rate, mean education for those aged 15 and older, and lag-distributed income per person, and computes the geometric mean of these three measures for each location (appendix p 17).^27^ The SDI for Indonesia in 2016 was 0·676. SDI was used to calculate expected mortality rates and YLDs by using Gaussian process regression with a linear prior for the mean function for each age-sex group. We compared these values with the observed values to identify locations and causes for which improvements were greater or less than anticipated on the basis of SDI alone.

### Role of the funding source

The funder of the study had no role in study design; collection, analysis, and interpretation of data; or writing of the report. The corresponding authors had full access to the data and had responsibility for final submission of the manuscript.

## Results

**Figure 1:**
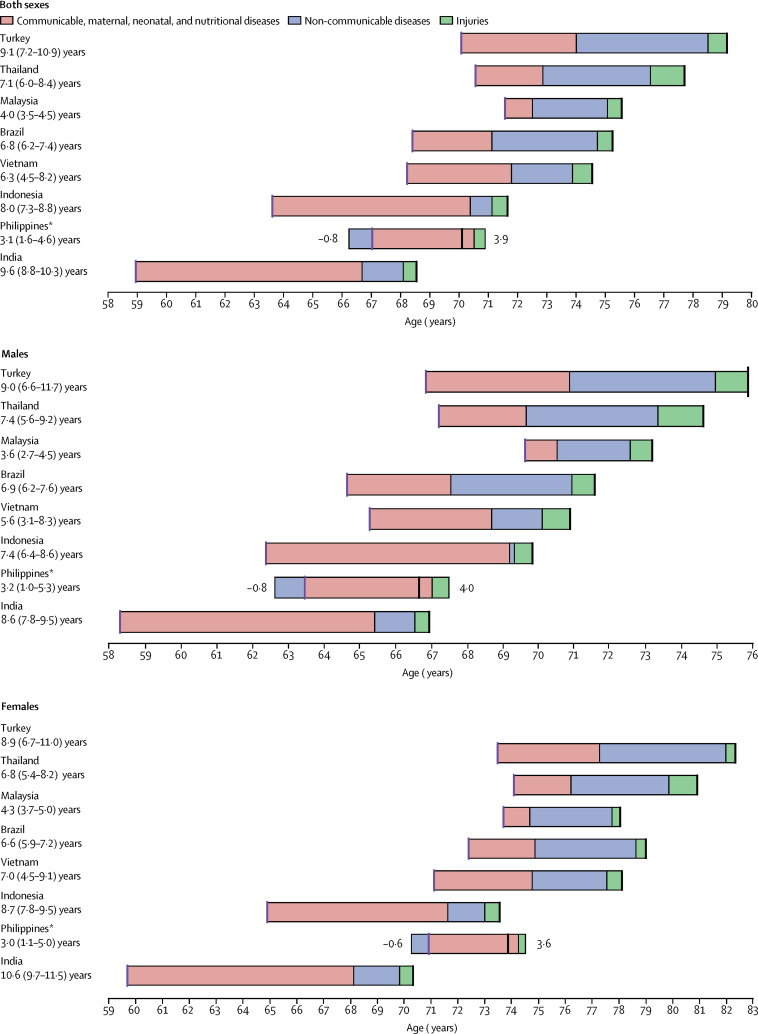
Attribution of changes in life expectancy at birth to changes in major groups of causes of death for Indonesia and comparator countries, 1990–2016, by sex Changes are shown for Indonesia and comparator countries for both sexes, males, and females. Locations are ordered by life expectancy at birth in 2016, from highest to lowest. Purple lines show life expectancy at birth in 1990, and black lines show life expectancy at birth in 2016; for all countries except the Philippines, these lie at the beginning and end of each bar, respectively. *In the Philippines, the increase in life expectancy attributable to changes in injuries and communicable, maternal, neonatal, and nutritional causes was countered by a decrease in life expectancy attributable to non-communicable diseases, leading to a smaller net increase in life expectancy than the overall change.

Between 1990 and 2016, life expectancy at birth in Indonesia increased by 8·0 years (95% UI 7·3–8·8), from 63·6 years (63·2–64·0) to 71·7 years (71·0–72·3; figure 1). Life expectancy at birth for males increased by 7·4 years (6·4–8·6), from 62·4 years (61·8–62·9) to 69·8 years (68·8–70·7), whereas life expectancy at birth for females increased by 8·7 years (7·8–9·5), from 64·9 years (64·3–65·4) to 73·6 years (73·0–74·1; figure 1).

For the same period, life expectancy at birth increased across the comparator countries on average by 6·6 years for both sexes, ranging from 9·6 years (95% UI 8·8–10·3) in India (from 59·0 years [58·5–59·4] to 68·6 years [67·9–69·1]) to 3·1 years (1·6–4·6) in the Philippines (from 67·0 years [66·4–67·7] to 70·1 years [68·7–71·5]).

**Figure 2:**
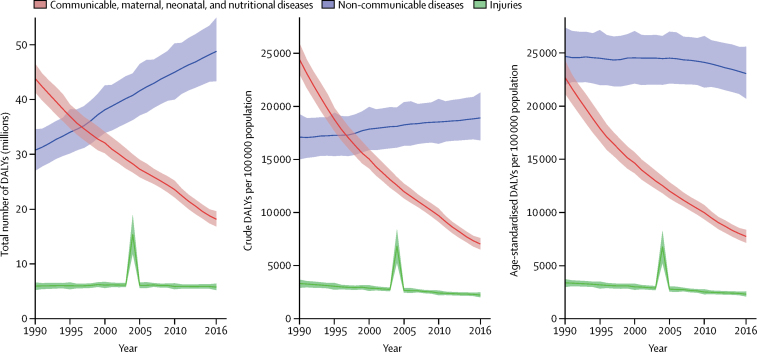
Trends in DALYs (total number, crude rates, and age-standardised rates) from 1990 to 2016 by GBD Level 1 cause groups: communicable, maternal, neonatal, and nutritional diseases; non-communicable diseases; and injuries The difference in trends between total DALYs and crude DALY rates is caused by population growth, and the difference between crude and age-standardised rates is caused by changes in the percentage distribution of the population by age. Shaded areas show 95% uncertainty intervals. DALYs=disability-adjusted life-years.

Between 1990 and 2016, Indonesia experienced a substantial decline in diseases due to communicable, maternal, neonatal, and nutritional (CMNN) causes: total CMNN DALYs declined by 58·6% (95% UI 55·6–61·6) from 43·8 million (95% UI 41·4–46·5) to 18·1 million (16·8–19·6). All-age rates and total CMNN DALYs dipped below those of NCDs in 1996, whereas total NCD DALYs rose substantially (figure 2). Age-standardised rates for CMNN DALYs also decreased substantially (by 65·9% [63·2–68·4]) from 1990 to 2016 (from 22·7 thousand [21·2–24·3] to 7·74 thousand [7·15–8·36]), whereas age-standardised rates for NCD DALYs remained stable across most of the study period but declined slightly from 2010 to 2016 (by 4·4% [2·6–6·2], from 24·1 thousand [21·7–26·6] to 23·1 thousand [20·7–25·6]). Total DALYs from injuries remained fairly stable across the study period, with the exception of the spike in 2004 corresponding to the Indian Ocean earthquake and tsunami. A substantial reduction was observed in crude rates (32·5% [23·5–39·0], from 3·33 thousand [2·94–3·70] to 2·25 thousand [2·04–2·50]), and in age-standardised rates (31·4% [23·4–37·2], from 3·40 thousand [3·01–3·75] to 2·33 thousand [2·12–2·59]).

**Figure 3:**
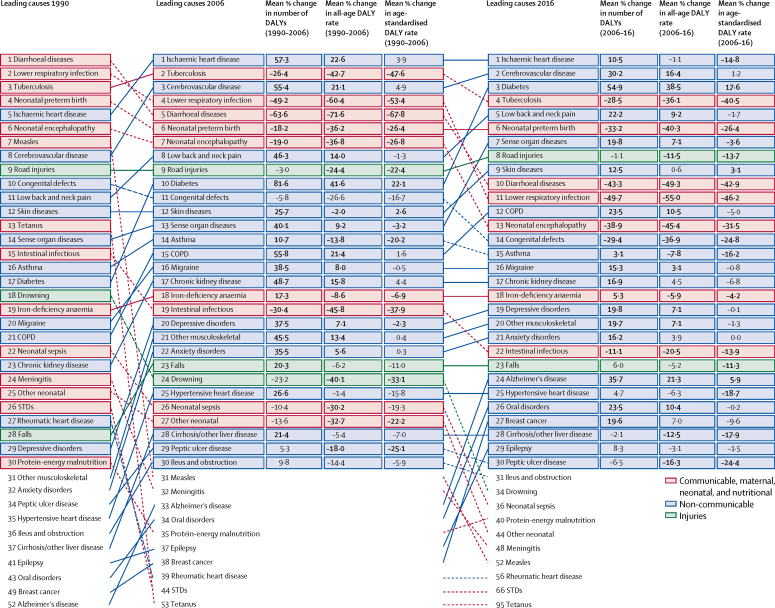
Leading 30 Level-3 causes of DALYs in Indonesia for 1990, 2006, and 2016, with percentage change in number of DALYs and all-age and age-standardised DALY rates Causes of DALYs for both sexes combined are ordered by total DALYs and are connected by arrows between time periods. For the time periods 1990–2006 and 2006–16, three measures of change are shown: median percent change in the number of DALYs, median percent change in the all-age DALY rate, and median percent change in the age-standardised DALY rate. Median values across the 1000 draws from the uncertainty distribution are shown. Numbers in bold are statistically significant (α=0·05). DALYs=disability-adjusted life-years. COPD=chronic obstructive pulmonary disease. STDs=sexually transmitted diseases. Cirrhosis/other liver disease=cirrhosis and other chronic liver diseases. Intestinal infectious=other intestinal infectious diseases.

In 1990, six of the ten leading causes of DALYs were CMNN conditions, which decreased to three of ten in 2016. Diarrhoeal diseases dropped from the number one cause of DALYs in 1990 to the fifth in 2006 (a total DALY reduction of 63·6% [95% UI 57·4–68·6], from 9·52 million [95% UI 7·39–11·9] to 3·45 million [2·72–4·27]), and dropped an additional 43·3% (29·1–52·0) from 2006 to 2016 to the tenth leading cause (1·95 million [1·48–2·51] total DALYs; figure 3). Lower respiratory infections likewise dropped from the second leading cause in 1990 to the 11th in 2016, representing a decrease of 74·4% (69·8–78·7) in total DALYs from 6·89 million (5·83–8·04) to 1·75 million (1·57–1·96). Tuberculosis remained among the leading causes of DALYs, moving from third in 1990 to second in 2006 (decrease in total DALYs of 26·4% [20·7–32·2], from 5·77 million [5·26–6·35] to 4·23 million [4·02–4·47]), and fourth in 2016 (decrease of 28·5% [24·0–32·4] to 3·03 million [2·84–3·23]). Neonatal preterm birth complications showed less dramatic reductions, moving from the fourth leading cause in 1990 to the sixth in 2006 (decrease of 18·2% [3·6–31·5], from 4·17 million [3·40–5·48] to 3·38 million [3·02–4·00], and remaining at sixth in 2016 (decrease of 33·2% [24·2–40·9], to 2·52 million [1·99–2·61]).

Non-communicable diseases comprised six of the leading ten causes of DALYs in 2016, compared with three in 1990 (figure 3). Ischaemic heart disease became the leading cause in 2006, with the total number of DALYs increasing by 57·3% (95% UI 44·5 to 70·0) between 1990 (3·76 million [95% UI 3·41 to 4·20]) and 2006 (5·90 million [5·63 to 6·22]). It remained the leading cause in 2016, having increased an additional 10·5% (5·43 to 16·3) between 2006 and 2016 to 6·52 million (6·18 to 6·88). DALYs from cerebrovascular disease also increased substantially between 1990 and 2006 (by 55·4% [46·3 to 66·3], from 2·56 million [2·37 to 2·75] to 3·98 million [3·80 to 4·17]) and 2006 and 2016 (by 30·2% [24·3 to 36·1], to 5·18 million [4·89 to 5·49]), rising from the eighth leading cause in 1990 to the second in 2016. Despite large increases in total DALYs, age-standardised rates for these conditions have remained stable; from 2006 to 2016, age-standardised rates of cerebrovascular disease increased by just 1·2% (−3·4 to 5·8) from 2·75 thousand (2·61 to 2·90) to 2·78 thousand (2·62 to 2·96), whereas age-standardised rates of ischaemic heart disease dropped by 14·8% (10·3 to 18·7), from 4·03 thousand (3·83 to 4·25) to 3·43 thousand (3·24 to 3·62). The exception to this pattern is diabetes, for which the number of DALYs increased between 2006 and 2016 by 54·9% (48·8 to 61·0), from 2·16 million (1·93 to 2·44) to 3·35 million (2·97 to 3·80), and age-standardised rates increased by 17·6% (12·8 to 22·4), from 1·35 thousand (1·21 to 1·50) to 1·58 thousand (1·41 to 1·77), making it the third leading cause of DALYs in 2016.

Road injuries moved from the ninth leading cause of DALYs in 1990 and 2006 to the eighth in 2016 (figure 3). Although the total number of DALYs from road injuries decreased over both time periods, they remain a leading cause of death and disability. Of the top causes of DALYs in 2016, road injuries and falls were the only injury-related causes to make the list.

**Figure 4:**
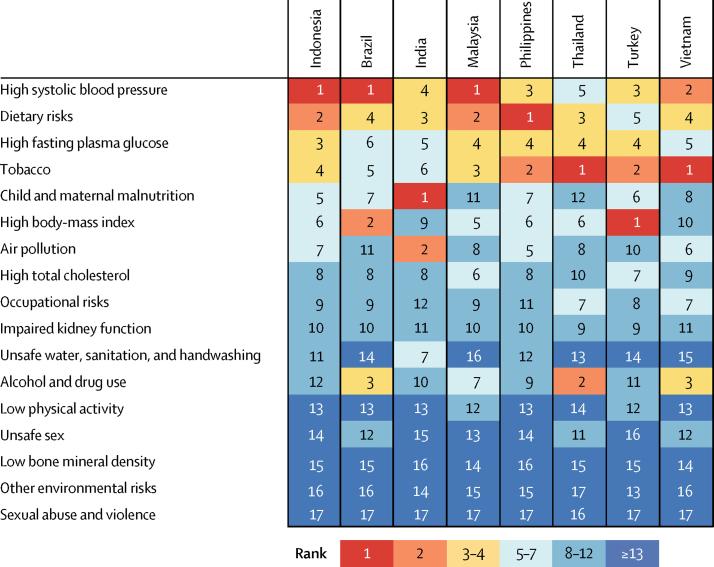
Ranking of age-standardised DALYs attributable to Level 2 risk factors in Indonesia and comparator countries in 2016 DALYs=disability-adjusted life-years.

When ranking risk factors by the number of age-standardised DALYs they contribute to in Indonesia and comparator countries in 2016, we found that the leading four risk factors in Indonesia also tended to be in the top three or four positions in comparator countries, showing a similar profile of health challenges (figure 4). The leading risk factor in Indonesia, high systolic blood pressure, ranks among the leading three causes in all comparator countries except India and Thailand (figure 4). Dietary risks are among the top five risk factors for all comparators, whereas high fasting plasma glucose is ranked higher in Indonesia than in the rest of the comparators (figure 4). Although tobacco is the fourth leading risk factor in Indonesia, it is among the top three risks in all comparators except Brazil and India (figure 4). Child and maternal malnutrition is ranked fifth in Indonesia, higher than in all the other comparator countries except India (figure 4).

**Figure 5:**
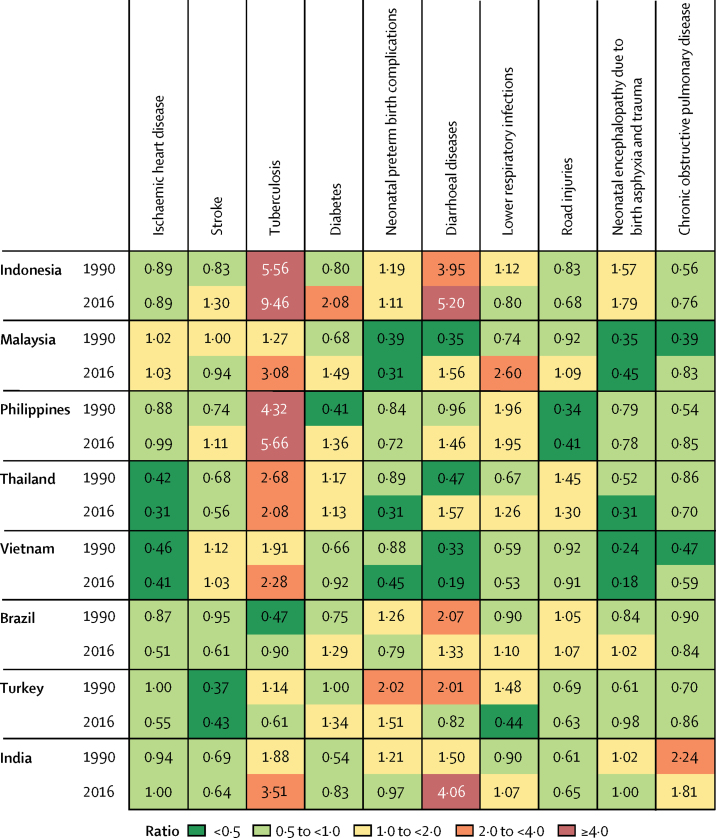
Ratio of observed to expected age-standardised rates of YLLs among ten leading causes of YLLs in Indonesia and comparator countries in 1990 and 2016 YLLs=years of life lost. COPD=chronic obstructive pulmonary disease.

The ratio of observed YLLs and the number of YLLs that would be expected on the basis of SDI alone increased in Indonesia between 1990 and 2016 for six of the ten leading causes of death (figure 5). The ratio for most leading causes of mortality in Indonesia was between 0·5 and 4·0, with the notable exceptions of diarrhoeal diseases and tuberculosis (figure 5). Three ratios decreased from 1990 to 2016: neonatal preterm birth complications, lower respiratory infections, and road injuries (figure 5). Similar patterns were seen among comparator countries: all comparators except Thailand and Turkey experienced an increase in the ratio for tuberculosis, although none resulting in a 2016 ratio as high as that of Indonesia (the closest being the Philippines; figure 5). Indonesia also had notably high and increasing ratios for diarrhoeal disease (figure 5).

**Figure 6:**
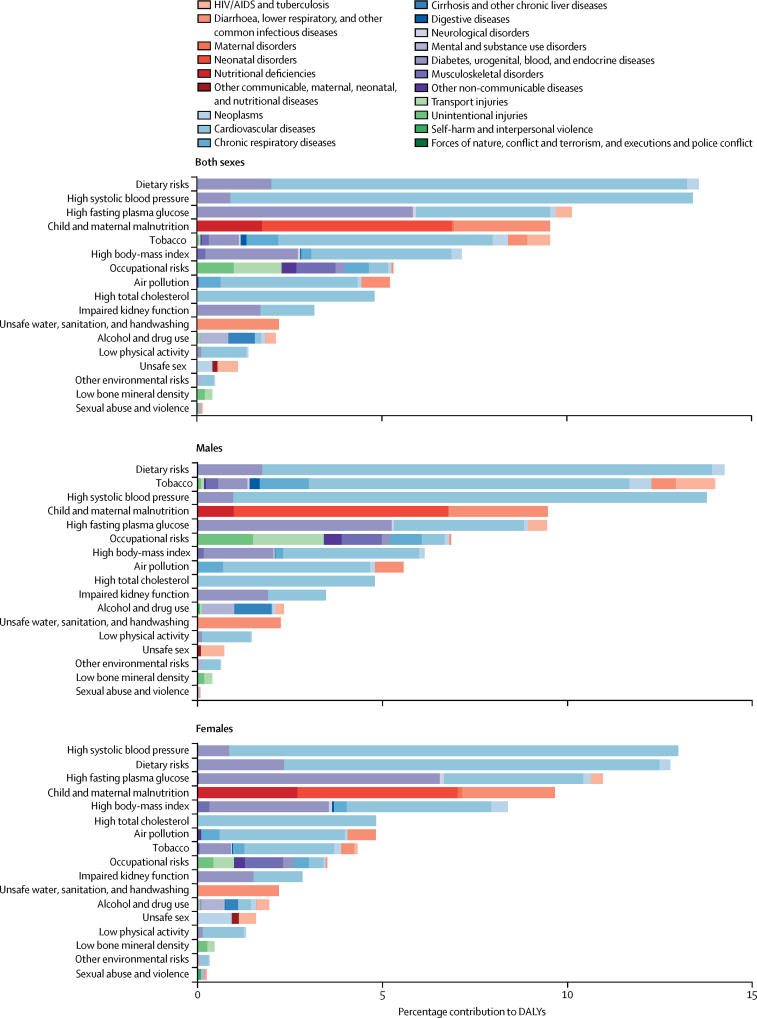
DALYs attributable to Level 2 risk factors in Indonesia in 2016, by sex DALYs=disability-adjusted life-years.

Dietary risks account for the highest number of DALYs (13·6% [95% UI 11·8–15·4]) in Indonesia, contributing substantially to the burden of cardiovascular disease (11·3% [9·7–12·8]); diabetes, urogenital, blood, and endocrine diseases (2·0% [1·5–2·6]); and, to a lesser degree, neoplasms (0·3% [0·2–0·4]; figure 6). High systolic blood pressure is the second leading risk factor, accounting for 13·4% (12·0–15·0) of DALYs and contributing principally to the burden of cardiovascular disease (12·5% [11·2–14·0]) and diabetes, urogenital, blood, and endocrine diseases (0·9% [0·8–1·1]). High fasting plasma glucose contributes to the third highest number of DALYs (10·1% [8·8–11·7]), including diabetes, urogenital, blood, and endocrine diseases (5·8% [5·5–6·1]); cardiovascular disease (3·6% [2·5–5·1]); and HIV/AIDS and tuberculosis (0·4% [0·3–0·6]). Other leading risk factors include tobacco, which caused 9·5% (8·6–10·6) of total DALYs, and child and maternal malnutrition, which contributed to 9·5% (8·7–10·7) of total DALYs. High body-mass index (7·2% [4· 7–9·9]), occupational risks (5·3% [4·9–5·7]), and air pollution (5·2% [4·6–5·9]) also contributed to a substantial portion of DALYs in 2016.

## Discussion

Life expectancy increased by approximately 8 years for Indonesians between 1990 and 2016. With this increase comes a changing age structure: 65% of the population is now of working age and the population aged 60 years and older is growing, projected to make up 12% of the population by 2025 and 16% by 2035.^28^ Maintaining quality of life for older Indonesians promises to be a growing challenge, with more than 25% of this age group already reporting at least one form of disability.^29^

At the same time, Indonesia must grapple with mixed and changing patterns of morbidity, mortality, and disability. These are further complicated by the size of the country, its diversity of urban and rural environments, varied levels of socioeconomic development, and a growing number of metropolitan conglomerations. The epidemiological transition has produced a double burden of disease in Indonesia, with the simultaneous increase of NCDs such as diabetes, cerebrovascular disease, and ischaemic heart disease, while CMNN causes such as tuberculosis, diarrhoea, and HIV/AIDS persist as substantial problems.

Based on comparisons between Indonesia and comparator nations, there is clearly potential for substantial improvements to health in Indonesia. Tuberculosis, neonatal preterm birth complications, diarrhoea, and lower respiratory tract infections are still substantial contributors to DALYs, and the rise in NCDs—especially ischaemic heart disease, cerebrovascular disease, and diabetes—needs increased attention.^30^ Diversity in treatment costs increases the complexity of addressing this double burden of disease. Road injuries, which cause the bulk of Indonesia's injury DALY burden, also present a substantial challenge. Improving personal and commercial transportation has been a high priority, yet increasing connectivity and safety demand ongoing attention from multiple branches of government.

The leading three risk factors for premature mortality—high systolic blood pressure, dietary risks, and high fasting plasma glucose—have an aetiological component that is directly or indirectly associated with diet and lifestyle. A 2015 analysis^31^ of the burden of malnutrition in Indonesia found a high prevalence of both undernutrition and overnutrition concentrated in specific clusters. In particular, the recent sharp rise of diabetes will continue to strain the health system. In response to these risks, the Indonesian Government has increased promotion and prevention measures, including Presidential Decree No. 1 (2017),^32^ to encourage healthy lifestyles, focusing on physical activity, healthy diet, and early detection of health problems. Additional reduction efforts through public health campaigns, taxation, and further legislation are required to ensure that the burden of these preventable risk factors does not continue to rise.

Ischaemic heart disease and haemorrhagic stroke are Indonesia's first and second leading causes of premature mortality. The dominant position of these causes reflects high levels of systolic blood pressure and smoking, which are major determinants of these conditions. Age-standardised DALY rates attributable to elevated systolic blood pressure in Indonesia were among the highest in the world in 2016, and the highest found outside of eastern and central Europe. DALY rates attributable to tobacco smoking were similarly high. These risks are particularly concerning in light of increasing numbers of incident stroke and myocardial infarctions, driven in large part by population growth and population ageing.

Tobacco is currently the fourth leading risk factor for premature death and disability in Indonesia, which also has the world's highest daily smoking rates for males: in 2016, more than half of males older than 10 years smoked daily.^25^ Although smoking rates among females are much lower, two thirds of Indonesian women are regularly exposed to second-hand smoke.^25^ Nonetheless, tobacco control remains highly contentious within the country, and Indonesia has yet to sign the WHO Framework Convention on Tobacco Control—the only country in Asia and one of only nine worldwide not to do so.^33^

Indonesian men and women differ in their health status in other ways as well: women had higher life expectancy at birth than did men and made greater gains in life expectancy from 1990 to 2016. The increase in life expectancy at birth in Indonesia was primarily due to reductions in CMNN diseases, although this proportion is higher in Indonesian males than in females, for whom a reduction in NCD causes contributed substantially more to increasing life expectancy at birth. Although diet-related risk factors were important for both sexes, smoking contributed to nearly the same percentage of DALYs in 2016 as did dietary risks for men, but contributed to a much smaller percentage of DALYs for women. These variances can guide targeted health interventions for both sexes.

Indonesia has 180·7 million people insured through JKN—approximately 70% of the population. Its target is to reach 95% enrolment in 2019.^34^ This programme will be key to sustaining health improvements, especially with appropriate actions to monitor, evaluate, and address widespread socioeconomic disparities in the supply and demand sides of the health system. Although increased government health spending and projected changes in JKN financing (from out-of-pocket to pooled resources) might positively impact inequality, concerns remain that too large a proportion of JKN funds have been directed to secondary-level services (ie, hospitalisation) and too little to preventive and primary care.^35^

The programme faces many challenges, including the complexity of devolved systems and the need for infrastructure improvement and human resources, alongside the paramount importance of maintaining support for primary health care. In addition, the health system must be able to respond to changing demand due to epidemiological shifts and diminishing financial barriers as a result of JKN. Ensuring that JKN is strong and sustainable is crucial to achieving universal health care in Indonesia.^36^, ^37^

The Universal Health Coverage Index, which measures progress toward the Sustainable Development Goal indicator of achieving universal coverage (SDG indicator 3.8) on a scale of 0–100, places Indonesia at 39.^37^ Indonesia's score shows that great progress remains to be made on service coverage. The country is expected to continue to improve substantially, with a projected index rating of 56 by 2030.

This Article is subject to all the limitations of the GBD methodology, which have been comprehensively described elsewhere.^21^, ^22^, ^25^, ^26^, ^37^, ^38^ Insufficiently comprehensive vital registration data is a challenge for many of the locations included in the GBD, and Indonesia is no exception. Most of the results presented for Indonesia come from regional patterns and covariates, with few datapoints available to guide estimation. Results could be strengthened by efforts to systematically fill this data gap. The move towards subnational GBD estimates is a key next step for Indonesia and will be uniquely important owing to the country's marked geographical, demographic, and cultural diversity, as well as the federated provincial-level authority in the health system. Additional urban–rural stratification of results will be highly beneficial both in terms of understanding disparities and directing appropriate health policy and programmes, and is particularly needed to target isolated populations living on the many small islands of Indonesia.

In conclusion, Indonesia has increased life expectancy at birth by 8 years since 1990, primarily through reductions in CMNN diseases. The archipelago is experiencing a double burden of disease at the national level: although changing lifestyles have rapidly increased prevalence of NCDs, there is still a large burden from CMNN diseases. The cost of health-care provision has risen sharply, and with it the cost and difficulty of reaching universal health care. This baseline study provides a better understanding of what risks remain to be targeted and the available opportunities for improvement. Given Indonesia's geographical and socioeconomic diversity, we will probably continue to see very diverse patterns of health and burden of disease across the country. Subnational estimates of the burden of disease will be particularly valuable in tailoring health priorities and programmes to the needs of specific provinces. The political commitment to the ongoing subnational GBD exercise is prudent and will increase the value of these metrics for both public health planning and assessment of progress over time.
