# Supplementary material for: On the road to universal health care in Indonesia, 1990–2016: a systematic analysis for the Global Burden of Disease Study 2016
Source: Lancet. 2018 Aug 18;392(10147):581–91. doi: 10.1016/S0140-6736(18)30595-6 (PMC6099123; doi:10.1016/S0140-6736(18)30595-6)

THE LANCET

Supplementary appendix

This appendix formed part of the original submission and has been peer reviewed. We post it as supplied by the authors.

Supplement to: Mboi N, Surbakti IM, Trihandini I, et al. On the road to universal health care in Indonesia, 1990–2016: a systematic analysis for the Global Burden of Disease Study 2016. *Lancet* 2018; published online June 28. http://dx.doi.org/10.1016/S0140-6736(18)30595-6.

Appendix

Contents

GATHER Table 2

Methods 16

1. Data 16
2. Mortality 16
3. Morbidity 16
4. Socio-demographic Index 17

Supplemental figures 17

SI Figure 1. Attribution of changes in under-5 mortality for Indonesia and comparator countries to changes in major groups of causes of death, 1990–2016 18

SI Figure 2. Leading 30 Level 3 causes of mortality in Indonesia for 1990, 2006, and 2016, with percent change in number of mortalities, and all-age and age-standardised mortality rates 21

SI Figure 3. Age-standardised rates of YLLs for males versus females in Indonesia and comparator countries, (A) 1990, (B) 2016 23

SI Figure 4. Leading 30 Level 3 causes of YLLs in Indonesia for 1990, 2006, and 2016, with percent change in number of YLLs, and all-age and age-standardised YLL rates 26

SI Figure 5. Leading 30 Level 3 causes of YLDs in Indonesia for 1990, 2006, and 2016, with percent change in number of YLDs, and all-age and age-standardised YLD rates 28

SI Figure 6. Leading 30 Level 3 risk factors for YLLs in Indonesia for 1990, 2006, and 2016, with percent change in number of YLLs, and all-age and age-standardised YLL rates 30

SI Figure 7. Ratio of observed to expected age-standardised YLLs in Indonesia and comparator countries, 1990 and 2016, with uncertainty intervals 32

Appendix Table 1. Guidelines for Accurate and Transparent Health Estimates Reporting (GATHER) checklist of information that should be included in reports of global health estimates, with description of compliance and location of information for "On the road to universal health care in Indonesia, 1990 to 2016: a systematic analysis for the Global Burden of Disease Study 2016."			
**#**	**GATHER checklist item**	**Description of Compliance**	**Reference**
**Objectives and funding**			
1	Define the indicators, populations, and time periods for which estimates were made.	Narrative provided in paper and methods appendix describing indicators, definitions, and populations	Main text (Methods) and methods appendix
2	List the funding sources for the work.	Funding sources listed in paper	Summary (Funding)
**Data Inputs**			
For all data inputs from multiple sources that are synthesized as part of the study:			
3	Describe how the data were identified and how the data were accessed.	Narrative description of data-seeking methods provided in the GBD 2016 publications* and methods appendices**	GBD 2016 Mortality appendix (pgs 6-9, 21-25, 53, 58-60), Cause of death appendix (pgs 9-13), Years lived with disabilities (YLDs) appendix (pgs 6-17)
4	Specify the inclusion and exclusion criteria. Identify all ad-hoc exclusions.	Narrative about inclusion and exclusion criteria by data type provided in the GBD 2016 publications* and methods appendices**	GBD Mortality appendix (pgs 6-9, 21-25, 53, 58-60) Cause of death appendix (by cause pgs 39-278), YLDs appendix (by cause pgs 34-716)
5	Provide information on all included data sources and their main characteristics. For each data source used, report reference information or contact name/institution, population represented, data collection method, year(s) of data collection, sex and age range, diagnostic criteria or measurement method, and sample size, as relevant.	An interactive, online data source tool that provides metadata for data sources by component, geography, cause, risk, or impairment has been developed	Online data citation tools http://ghdx.healthdata.org/

6	Identify and describe any categories of input data that have potentially important biases (eg, based on characteristics listed in item 5).	Summary of known biases by cause provided in the GBD 2016 publications* and methods appendices **	Main Text, Limitations section in GBD Mortality, Cause of death, and YLD papers<br><br>Cause of death appendix (by cause pgs 39-278), YLDs appendix (by cause pgs 34-716)
For data inputs that contribute to the analysis but were not synthesised as part of the study:			
7	Describe and give sources for any other data inputs.	Included in online data source tool	Online data citation tools http://ghdx.healthdata.org/
For all data inputs:			
8	Provide all data inputs in a file format from which data can be efficiently extracted (eg, a spreadsheet as opposed to a PDF), including all relevant metadata listed in item 5. For any data inputs that cannot be shared due to ethical or legal reasons, such as third-party ownership, provide a contact name or the name of the institution that retains the right to the data.	Downloads of input data available through online tools, including data visualisation tools and data query tools; input data not available in tools will be made available upon request	Online data visualisation tools, data query tools, and the Global Health Data Exchange www.healthdata.org/results/data-visualizations; http://ghdx.healthdata.org/gb-results-tool; http://ghdx.healthdata.org/
**Data analysis**			
9	Provide a conceptual overview of the data analysis method. A diagram may be helpful.	Flow diagrams of the overall methodological processes, as well as cause-specific modelling processes, have been provided in the GBD 2016 publications* and methods appendices**	GBD Mortality appendix figure 1, Cause of death appendix figures 1-3, YLDs appendix figures 1-2

10	Provide a detailed description of all steps of the analysis, including mathematical formulae. This description should cover, as relevant, data cleaning, data pre-processing, data adjustments and weighting of data sources, and mathematical or statistical model(s).	Flow diagrams and corresponding methodological write-ups for each cause, as well as the demographics and causes of death databases and modelling processes, have been provided in the GBD 2016 publications* and methods appendices**	GBD Mortality appendix figure 1, Cause of death appendix figures 1-3, YLDs appendix figures 1-2<br><br>GBD Cause of death appendix (by cause pgs 39-278), YLDs appendix (by cause pgs 34-716)
11	Describe how candidate models were evaluated and how the final model(s) were selected.	Provided in the methodological write-ups in the GBD 2016 publications* and methods appendices **	GBD Cause of death appendix (by cause pgs 39-278), YLDs appendix (by cause pgs 34-716)
12	Provide the results of an evaluation of model performance, if done, as well as the results of any relevant sensitivity analysis.	Provided in the methodological write-ups in the GBD 2016 publications* and methods appendices **	GBD Cause of death appendix (by cause pgs 39-278), YLDs appendix (by cause pgs 34-716)
13	Describe methods for calculating uncertainty of the estimates. State which sources of uncertainty were, and were not, accounted for in the uncertainty analysis.	Narrative provided in the main paper<br><br>Further detail available in the GBD 2016 publications* and methods appendices**	Main text (Methods)<br><br>GBD Cause of death appendix (pgs 9, 33-35), YLD appendix (pgs 10, 28-29)
14	State how analytic or statistical source code used to generate estimates can be accessed.	Access statement provided in methods	Main text (Methods)<br><br>Code is provided in an online repository https://github.com/ihmeuw/ihme-modeling

**Results and Discussion**			
15	Provide published estimates in a file format from which data can be efficiently extracted.	GBD 2016 results are available through online data visualisation tools, the Global Health Data Exchange, and the online data query tool	Main text and online data tools (data visualisation tools, data query tools, and the Global Health Data Exchange) www.healthdata.org/results/data-visualizations; http://ghdx.healthdata.org/gb-results-tool; http://ghdx.healthdata.org/
16	Report a quantitative measure of the uncertainty of the estimates (eg, uncertainty intervals).	Uncertainty intervals are provided with all results	Main text (Methods, results), methods appendix and online data tools (data visualisation tools, data query tools, and the Global Health Data Exchange) www.healthdata.org/results/data-visualizations; http://ghdx.healthdata.org/gb-results-tool; http://ghdx.healthdata.org/
17	Interpret results in light of existing evidence. If updating a previous set of estimates, describe the reasons for changes in estimates.	Discussion of methodological changes between GBD rounds provided in the research in context section of the paper	Research in context
18	Discuss limitations of the estimates. Include a discussion of any modelling assumptions or data limitations that affect interpretation of the estimates.	Discussion of limitations provided in the narrative of the paper	Main text (Discussion, limitations)

**Gakidou E, Afshin A, Abajobir AA, et al. Global, regional, and national comparative risk assessment of 84 behavioural, environmental and occupational, and metabolic risks or clusters of risks, 1990–2016: a*

systematic analysis for the Global Burden of Disease Study 2016. The Lancet 2017; 390: 1345–422.

***Supplementary appendix 1*

**Hay SI, Abajobir AA, Abate KH, et al. Global, regional, and national disability-adjusted life-years (DALYs) for 333 diseases and injuries and healthy life expectancy (HALE) for 195 countries and territories, 1990–2016: a systematic analysis for the Global Burden of Disease Study 2016. The Lancet 2017; 390: 1260–344.*

*** Supplementary appendix 1*

**Naghavi M, Abajobir AA, Abbafati C, et al. Global, regional, and national age-sex specific mortality for 264 causes of death, 1980–2016: a systematic analysis for the Global Burden of Disease Study 2016. The Lancet 2017; 390: 1151–210.*

*** Supplementary appendix 1*

**Vos T, Abajobir AA, Abate KH, et al. Global, regional, and national incidence, prevalence, and years lived with disability for 328 diseases and injuries for 195 countries, 1990–2016: a systematic analysis for the Global Burden of Disease Study 2016. The Lancet 2017; 390: 1211–59.*

*** Supplementary appendix 1*

**Wang H, Abajobir AA, Abate KH, et al. Global, regional, and national under-5 mortality, adult mortality, age-specific mortality, and life expectancy, 1970–2016: a systematic analysis for the Global Burden of Disease Study 2016. The Lancet 2017; 390: 1084–150.*

*** Supplementary appendix 1*

Appendix Table 2. Total number of site years* by cause and source type, cause of death estimates 1990-2016 Indonesia								
Level	Cause	Vital Registration	Verbal Autopsy	Surveillance	Sibling History	Survey/Census	Cancer Registry	Police Records
0	All causes		150		805	233		3
1	Communicable, maternal, neonatal, and nutritional diseases		134		805	214		
2	HIV/AIDS and tuberculosis		113					
3	Tuberculosis		110					
4	Drug-susceptible tuberculosis							
4	Multidrug-resistant tuberculosis without extensive drug resistance							
4	Extensively drug-resistant tuberculosis							
3	HIV/AIDS		83					
4	Drug-susceptible HIV/AIDS - Tuberculosis							
4	Multidrug-resistant HIV/AIDS - Tuberculosis without extensive drug resistance							
4	Extensively drug-resistant HIV/AIDS - Tuberculosis							
4	HIV/AIDS resulting in other diseases							
2	Diarrhea, lower respiratory, and other common infectious diseases		115					
3	Diarrheal diseases		104					
3	Intestinal infectious diseases		90					
4	Typhoid fever							
4	Paratyphoid fever							
4	Other intestinal infectious diseases							
3	Lower respiratory infections		105					
3	Upper respiratory infections							
3	Otitis media		56					
3	Meningitis		85					
4	Pneumococcal meningitis							
4	*H influenzae* type B meningitis							
4	Meningococcal meningitis							
4	Other meningitis							
3	Encephalitis		83					
3	Diphtheria							
3	Whooping cough							

3	Tetanus		76					
3	Measles		58					
3	Varicella and herpes zoster							
2	Neglected tropical diseases and malaria		96					
3	Malaria		81					
3	Chagas disease							
3	Leishmaniasis		67					
4	Visceral leishmaniasis		67					
3	African trypanosomiasis		63					
3	Schistosomiasis							
3	Cysticercosis							
3	Cystic echinococcosis							
3	Dengue		80					
3	Yellow fever		69					
3	Rabies		71					
3	Intestinal nematode infections		70					
4	Ascariasis							
3	Ebola virus disease							
3	Zika virus disease							
3	Other neglected tropical diseases							
2	Maternal disorders		64		805	187		
3	Maternal haemorrhage		38					
3	Maternal sepsis and other maternal infections		28					
3	Maternal hypertensive disorders		42					
3	Maternal obstructed labor and uterine rupture		21					
3	Maternal abortion, miscarriage, and ectopic pregnancy		32					
3	Indirect maternal deaths		31					
3	Late maternal deaths							
3	Maternal deaths aggravated by HIV/AIDS		64		805	187		
3	Other maternal disorders							
2	Neonatal disorders		59					
3	Neonatal preterm birth complications		44					

3	Neonatal encephalopathy due to birth asphyxia and trauma		45						
3	Neonatal sepsis and other neonatal infections								
3	Hemolytic disease and other neonatal jaundice								
3	Other neonatal disorders								
2	Nutritional deficiencies		77						
3	Protein-energy malnutrition		74						
3	Iodine deficiency								
3	Iron-deficiency anemia		56						
3	Other nutritional deficiencies								
2	Other communicable, maternal, neonatal, and nutritional diseases		97						
3	Sexually transmitted diseases excluding HIV		75						
4	Syphilis								
4	Chlamydial infection								
4	Gonococcal infection								
4	Other sexually transmitted diseases								
3	Hepatitis		84						
4	Acute hepatitis A								
4	Hepatitis B								
4	Hepatitis C								
4	Acute hepatitis E								
3	Other infectious diseases		87						
1	Non-communicable diseases		135						
2	Neoplasms		108						
3	Lip and oral cavity cancer		70						
3	Nasopharynx cancer								
3	Other pharynx cancer								
3	Esophageal cancer		68						
3	Stomach cancer								
3	Colon and rectum cancer		78						
3	Liver cancer								
4	Liver cancer due to hepatitis B								
4	Liver cancer due to hepatitis C								

4	Liver cancer due to alcohol use							
4	Liver cancer due to other causes							
3	Gallbladder and biliary tract cancer							
3	Pancreatic cancer							
3	Larynx cancer							
3	Tracheal, bronchus, and lung cancer		70					
3	Malignant skin melanoma							
3	Non-melanoma skin cancer							
4	Non-melanoma skin cancer (squamous-cell carcinoma)							
3	Breast cancer		74					
3	Cervical cancer							
3	Uterine cancer							
3	Ovarian cancer							
3	Prostate cancer							
3	Testicular cancer		40					
3	Kidney cancer							
3	Bladder cancer							
3	Brain and nervous system cancer							
3	Thyroid cancer							
3	Mesothelioma							
3	Hodgkin's lymphoma							
3	Non-Hodgkin lymphoma							
3	Multiple myeloma							
3	Leukaemia		78					
4	Acute lymphoid leukaemia							
4	Chronic lymphoid leukaemia							
4	Acute myeloid leukaemia							
4	Chronic myeloid leukaemia							
4	Other leukaemia		78					
3	Other neoplasms							
2	Cardiovascular diseases		123					
3	Rheumatic heart disease							
3	Ischaemic heart disease		103					
3	Cerebrovascular disease		106					

4	Ischaemic stroke							
4	Haemorrhagic stroke							
3	Hypertensive heart disease							
3	Cardiomyopathy and myocarditis							
4	Myocarditis							
4	Alcoholic cardiomyopathy							
4	Other cardiomyopathy							
3	Atrial fibrillation and flutter							
3	Aortic aneurysm							
3	Peripheral artery disease							
3	Endocarditis							
3	Other cardiovascular and circulatory diseases							
2	Chronic respiratory diseases		109					
3	Chronic obstructive pulmonary disease							
3	Pneumoconiosis							
4	Silicosis							
4	Asbestosis							
4	Coal workers pneumoconiosis							
4	Other pneumoconiosis							
3	Asthma							
3	Interstitial lung disease and pulmonary sarcoidosis							
3	Other chronic respiratory diseases							
2	Cirrhosis and other chronic liver diseases		97					
3	Cirrhosis and other chronic liver diseases due to hepatitis B							
3	Cirrhosis and other chronic liver diseases due to hepatitis C							
3	Cirrhosis and other chronic liver diseases due to alcohol use							
3	Cirrhosis and other chronic liver diseases due to other causes							
2	Digestive diseases		102					
3	Peptic ulcer disease							
3	Gastritis and duodenitis							
3	Appendicitis							

3	Paralytic ileus and intestinal obstruction		67					
3	Inguinal, femoral, and abdominal hernia		55					
3	Inflammatory bowel disease							
3	Vascular intestinal disorders							
3	Gallbladder and biliary diseases							
3	Pancreatitis							
3	Other digestive diseases							
2	Neurological disorders		65					
3	Alzheimer's disease and other dementias							
3	Parkinson's disease							
3	Epilepsy		38					
3	Multiple sclerosis							
3	Motor neuron disease							
3	Other neurological disorders							
2	Mental and substance use disorders		46					
3	Alcohol use disorders		46					
3	Drug use disorders		43					
4	Opioid use disorders							
4	Cocaine use disorders							
4	Amphetamine use disorders							
4	Other drug use disorders							
3	Eating disorders							
4	Anorexia nervosa							
4	Bulimia nervosa							
2	Diabetes, urogenital, blood, and endocrine diseases		111					
3	Diabetes mellitus		96					
3	Acute glomerulonephritis							
3	Chronic kidney disease		102					
4	Chronic kidney disease due to diabetes mellitus							
4	hypertension							
4	Chronic kidney disease due to glomerulonephritis							
4	causes							
3	Urinary diseases and male infertility		84					

4	Interstitial nephritis and urinary tract infections							
4	Urolithiasis							
4	Other urinary diseases							
3	Gynecological diseases		51					
4	Uterine fibroids							
4	Polycystic ovarian syndrome							
4	Endometriosis							
4	Genital prolapse							
4	Other gynaecological diseases							
3	Haemoglobinopathies and hemolytic anaemias		90					
4	Thalassaemias		87					
4	Sickle cell disorders		87					
4	G6PD deficiency							
4	Other haemoglobinopathies and hemolytic anaemias							
3	Endocrine, metabolic, blood, and immune disorders		88					
2	Musculoskeletal disorders							
3	Rheumatoid arthritis							
3	Other musculoskeletal disorders							
2	Other non-communicable diseases		97					
3	Congenital birth defects		82					
4	Neural tube defects							
4	Congenital heart anomalies							
4	Orofacial clefts							
4	Down syndrome							
4	Other chromosomal abnormalities							
4	Congenital musculoskeletal and limb anomalies							
4	Urogenital congenital anomalies							
4	Digestive congenital anomalies							
4	Other congenital birth defects							
3	Skin and subcutaneous diseases		85					
4	Cellulitis							
4	Pyoderma							

4	Decubitus ulcer							
4	Other skin and subcutaneous diseases							
3	Sudden infant death syndrome							
1	Injuries		117			233		3
2	Transport injuries		108			229		1
3	Road injuries							1
4	Pedestrian road injuries							1
4	Cyclist road injuries							1
4	Motorcyclist road injuries							1
4	Motor vehicle road injuries							1
4	Other road injuries							1
3	Other transport injuries							
2	Unintentional injuries		102					
3	Falls		90					
3	Drowning		76					
3	Fire, heat, and hot substances		74					
3	Poisonings		64					
3	Exposure to mechanical forces		81					
4	Unintentional firearm injuries		70					
4	Unintentional suffocation		52					
4	Other exposure to mechanical forces		80					
3	Adverse effects of medical treatment							
3	Animal contact		65					
4	Venomous animal contact		55					
4	Non-venomous animal contact							
3	Foreign body		79					
4	Pulmonary aspiration and foreign body in airway							
4	Foreign body in other body part							
3	Environmental heat and cold exposure		65					
3	Other unintentional injuries		84					
2	Self-harm and interpersonal violence		88					2
3	Self-harm		77					
4	Self-harm by firearm							
4	Self-harm by other specified means							

3	Interpersonal violence		79					2
4	Physical violence by firearm		64					
4	Physical violence by sharp object		73					
4	Physical violence by other means		70					
2	Forces of nature, conflict and terrorism, and executions and police conflict		59					
3	Exposure to forces of nature		54					
3	Conflict and terrorism		56					
3	Executions and police conflict		49					

*A site year for a disease indicates that data were collected on that disease, not that there were incident cases in that site year. Some diseases may have site year data but zero cases of disease.

Methods

The GBD 2016 study organises causes of mortality and morbidity within a four-level classification hierarchy to produce estimates that are mutually exclusive and collectively exhaustive. The full GBD cause hierarchy, including corresponding International Classification of Diseases (ICD)-9 and ICD-10 codes, is detailed in the respective GBD 2016 publications.[17,18] Risk factors are likewise organised in a four-tier hierarchy.[19] Additional details on GBD metrics and definitions are found elsewhere.[20]

1. Data

Table 2 shows the data sources included in the estimation process from Indonesia for mortality and morbidity by cause. For GBD 2016 a star ranking system from 0 to 5 was developed to assess data quality in each location. Indonesia received 2 stars in 2016, with 14.5% well certified data for the period 1980-2016 and 56.7% for 2010-2016.

2. Mortality

We report data on cause-specific mortality and cause-specific years of life lost due to premature mortality (years of life lost, YLLs) for 264 causes of mortality by age and sex.[17,22] Cause-specific mortality estimates for each age, sex, and location-year were generated using the GBD Cause of Death Ensemble model (CODEm) and the CodCorrect process. In CODEm, a variety of models were developed. For each individual model, out-of-sample predictive validity was assessed and models were ranked for use in ensemble modelling. The ensemble with the highest out-of-sample predictive validity was selected from differently weighted combinations of individual models. For causes where there was evidence that children and adults had different relationships between their covariates and death rates, separate models were run for different age ranges. Separate models were also developed for countries with complete, representative, and extensive VR by cause to ensure that uncertainty reflects the more complete data in those locations. The CoDCorrect process ensures that there is internal consistency between cause-specific and all-cause mortality estimates by rescaling causes up the GBD hierarchy using a core algorithm.

3. Morbidity

Estimates of morbidity were produced for 328 causes and 2,982 sequela for each age, sex, and year. For GBD 2016, 68 causes were considered causes of disability but not death, while five were considered causes of death but not disability. To estimate years of life lived with disability (YLDs), the Bayesian meta-regression tool DisMod-MR 2.1 estimated prevalence and incidence for most causes of disease and injury and their non-fatal outcomes, calculated the product of incidence and a specific disability weight for each sequela, adjusted for comorbidity, and aggregated to cause-level, ensuring consistency for each condition. Several causes were estimated using custom models; details of these causes and their modelling strategy can be found in the GBD YLDs publication. YLDs were estimated for all mutually exclusive sequelae by multiplying prevalence by a disability weight, then correcting for comorbidity and aggregating to cause level.

4. SDI

The SDI is a summary indicator based on average lag-dependent income per capita, total fertility rate in the population, and years of education attained in the population over 15 years of age, calculated as the geometric mean of the rescaled (0-1) values of the three components for each location-year. GBD 2016 grouped locations into quintiles of low, low-middle, middle, high-middle, and high SDI for analysis.

Expected values calculation

Gaussian process regression using a linear prior to the mean function within a stochastic partial differential equation (SPDE) framework was used to estimate the relationship between SDI and each age-sex-cause death rate. These relationships were used to estimate expected YLLs based on SDI alone for each age, sex, location, and year.

Scale of SDI

For each component of the SDI, 0 represents a theoretical minimum level of development for the selected health outcomes and 1 represents a theoretical maximum level of development for the selected health outcomes. Thresholds were set based on the relationship between each component with under-5 mortality rates and life expectancy at birth and identified points of limiting returns if they occurred prior to theoretical limits.

Supplemental figures

SI Figure 1. Attribution of changes in under-5 mortality for Indonesia and comparator countries to changes in major groups of causes of death, 1990–2016

Changes are shown for Indonesia and comparator countries for both sexes combined. Locations are ordered by decreasing under-5 mortality in 2016. Purple lines show under-5 mortality rate in 1990 and black lines show under-5 mortality rate in in 2016. Causes to the left of the 1990 under-5 mortality values reflect causes that contributed to reductions in under-5 mortality rate from 1990 to 2015. Causes to the right of the 1990 under-5 mortality values contributed to increases in under-5 mortality rate from 1990 to 2015.
CMNN=communicable, maternal, neonatal & nutritional causes. NCDs=non-communicable diseases.

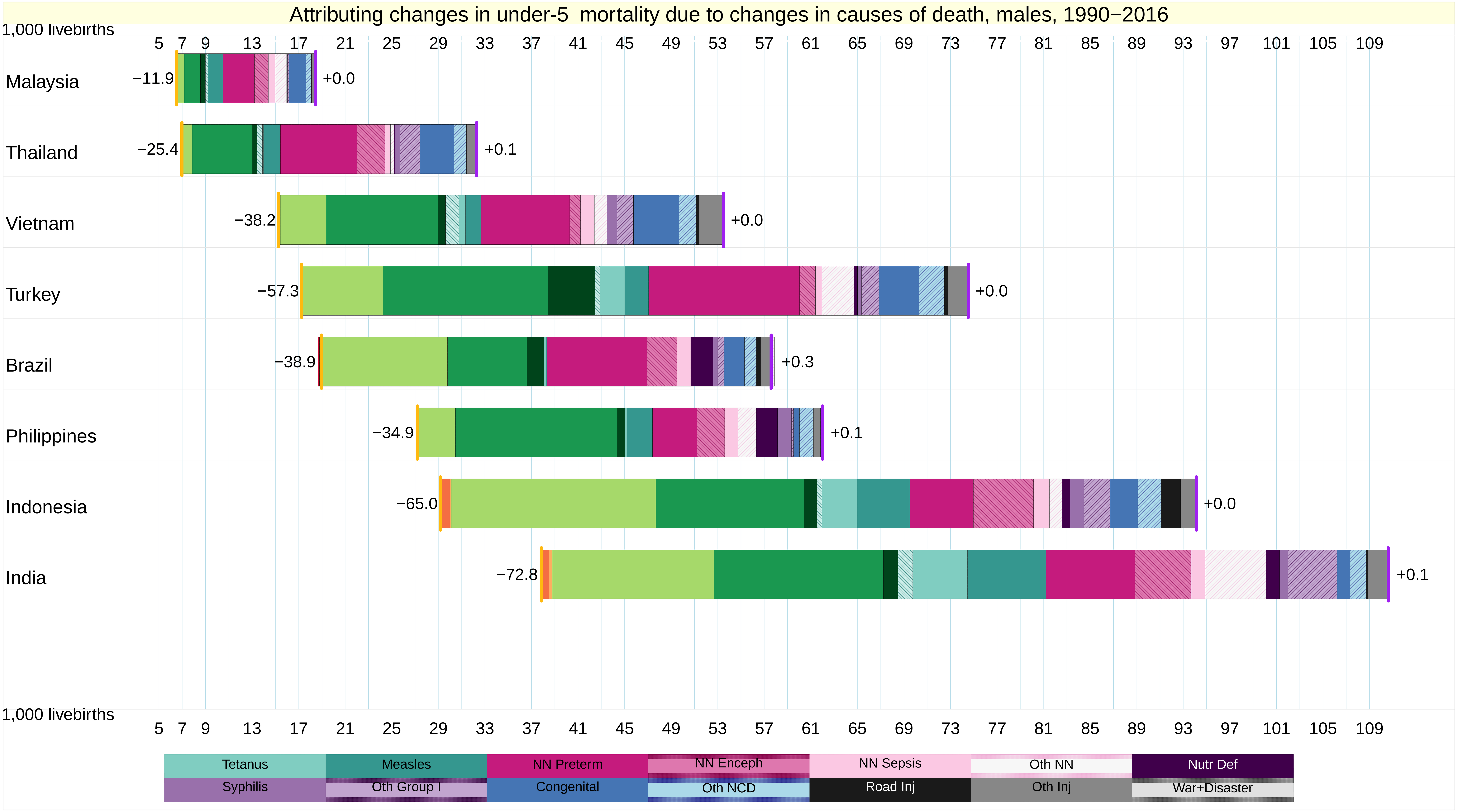

Attributing changes in under-5 mortality due to changes in causes of death, males, 1990−2016
1,000 livebirths
5 7 9 13 17 21 25 29 33 37 41 45 49 53 57 61 65 69 73 77 81 85 89 93 97 101 105 109
Malaysia
−11.9
+0.0
Thailand
−25.4
+0.1
Vietnam
−38.2
+0.0
Turkey
−57.3
+0.0
Brazil
−38.9
+0.3
Philippines
−34.9
+0.1
Indonesia
−65.0
+0.0
India
−72.8
+0.1
1,000 livebirths
5 7 9 13 17 21 25 29 33 37 41 45 49 53 57 61 65 69 73 77 81 85 89 93 97 101 105 109
Tetanus
Measles
NN Preterm
NN Enceph
NN Sepsis
Oth NN
Nutr Def
Syphilis
Oth Group I
Congenital
Oth NCD
Road Inj
Oth Inj
War+Disaster

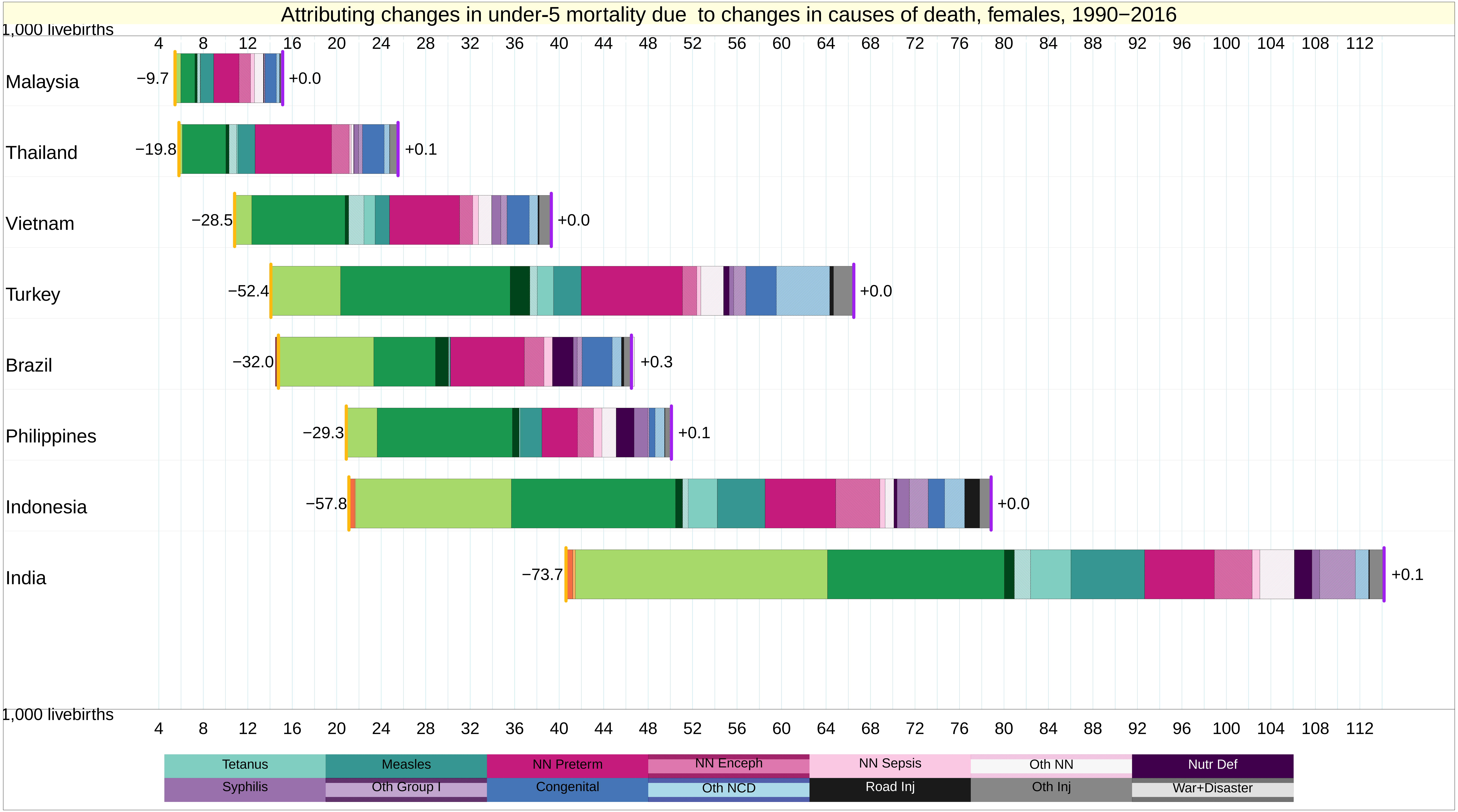

Attributing changes in under-5 mortality due to changes in causes of death, females, 1990−2016
1,000 livebirths
4 8 12 16 20 24 28 32 36 40 44 48 52 56 60 64 68 72 76 80 84 88 92 96 100 104 108 112
Malaysia
−9.7
+0.0
Thailand
−19.8
+0.1
Vietnam
−28.5
+0.0
Turkey
−52.4
+0.0
Brazil
−32.0
+0.3
Philippines
−29.3
+0.1
Indonesia
−57.8
+0.0
India
−73.7
+0.1
1,000 livebirths
4 8 12 16 20 24 28 32 36 40 44 48 52 56 60 64 68 72 76 80 84 88 92 96 100 104 108 112
Tetanus
Measles
NN Preterm
NN Enceph
NN Sepsis
Oth NN
Nutr Def
Syphilis
Oth Group I
Congenital
Oth NCD
Road Inj
Oth Inj
War+Disaster

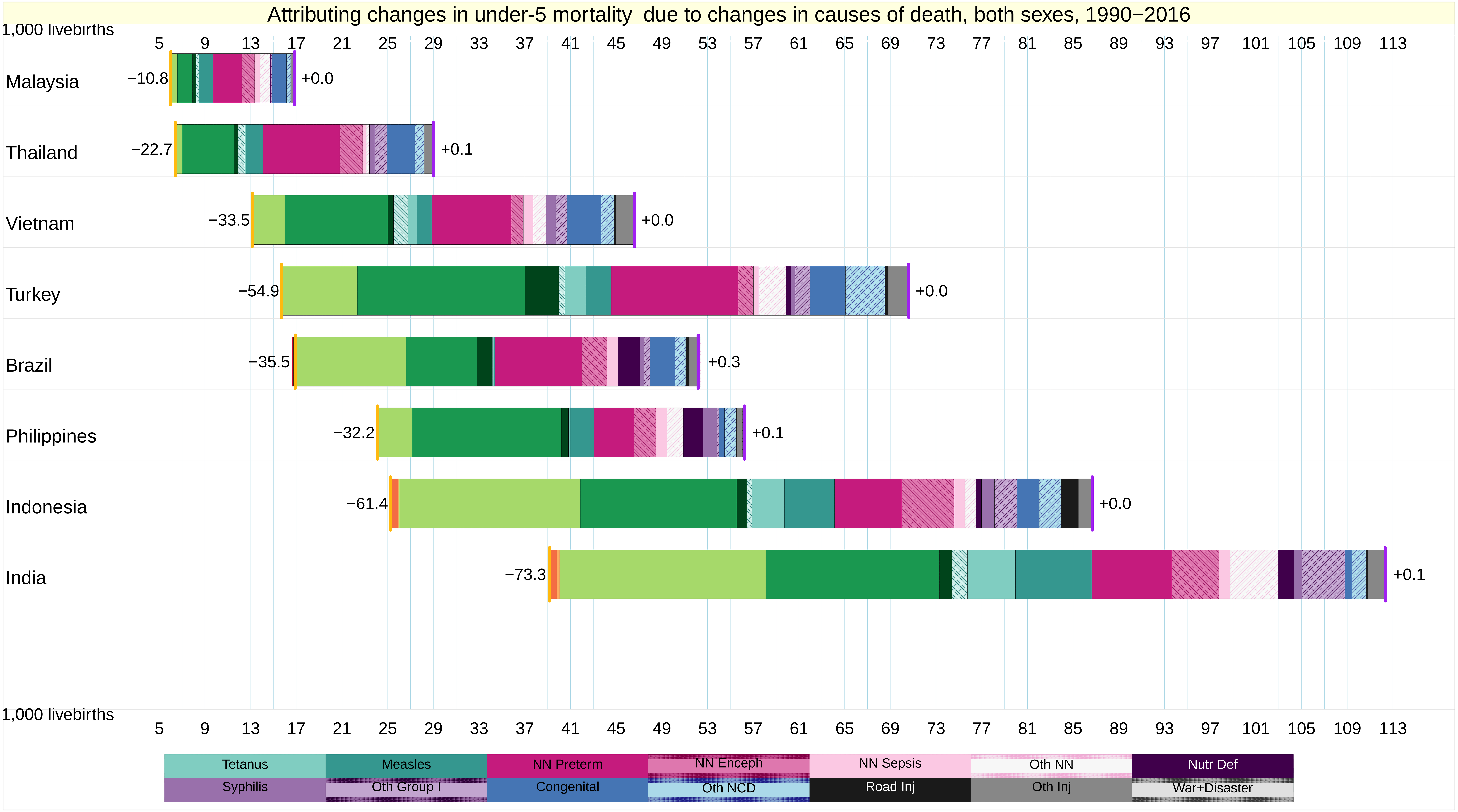
Attributing changes in under-5 mortality due to changes in causes of death, both sexes, 1990−2016
1,000 livebirths
5 9 13 17 21 25 29 33 37 41 45 49 53 57 61 65 69 73 77 81 85 89 93 97 101 105 109 113
Malaysia
−10.8
+0.0
Thailand
−22.7
+0.1
Vietnam
−33.5
+0.0
Turkey
−54.9
+0.0
Brazil
−35.5
+0.3
Philippines
−32.2
+0.1
Indonesia
−61.4
+0.0
India
−73.3
+0.1
1,000 livebirths
5 9 13 17 21 25 29 33 37 41 45 49 53 57 61 65 69 73 77 81 85 89 93 97 101 105 109 113
Tetanus
Measles
NN Preterm
NN Enceph
NN Sepsis
Oth NN
Nutr Def
Syphilis
Oth Group I
Congenital
Oth NCD
Road Inj
Oth Inj
War+Disaster

SI Figure 2. Leading 30 Level 3 causes of mortality in Indonesia for 1990, 2006, and 2016, with percent change in number of mortalities, and all-age and age-standardised mortality rates

Causes are ordered by total deaths and are connected by arrows between time periods. Communicable, maternal, neonatal, and nutritional disorders are shown in red, non-communicable causes in blue, and injuries in green. For the time period 1990 to 2006 and for 2006 to 2015, three measures of change are shown: median percent change in the number of mortalities, median percent change in the all-age mortality rate, and median percent change in the age-standardised mortality rate. Median values across the 1,000 draws from the uncertainty distribution are shown. Both sexes combined. COPD=chronic obstructive pulmonary disease.

Top 30 Level 3 causes of deaths for 1990, 2006, and 2016, Indonesia, both sexes

Causes are connected by arrows between time periods. Communicable, maternal, neonatal, and nutritional disorders are shown in red, non-communicable causes in blue and injuries in green. For the time period 1990 to 2016, three measures of change are shown: percent change in the number of deaths, percent change in the all-age death rate, and percent change in the age-standardised death rate. Numbers in bold highlight statistically significant changes.

Leading causes 1990
1 Diarrhoeal diseases
2 Tuberculosis
3 Ischaemic heart disease
4 Cerebrovascular disease
5 Lower respiratory infections
6 Neonatal preterm birth complications
7 Road injuries
8 Neonatal encephalopathy
9 Measles
10 Diabetes
11 Asthma
12 COPD
13 Congenital defects
14 Hypertensive heart disease
15 Tetanus
16 Peptic ulcer disease
17 Chronic kidney disease
18 Intestinal infectious diseases
19 Alzheimer's disease
20 Rheumatic heart disease
21 Falls
22 Drowning
23 Protein-energy malnutrition
24 Cirrhosis other
25 Ileus & obstruction
26 Other cardiovascular diseases
27 Malaria
28 Neonatal sepsis
29 Breast cancer
30 Meningitis
31 Stomach cancer
33 Cirrhosis due to hepatitis B
37 Lung cancer
38 Cirrhosis due to hepatitis C
39 Cirrhosis due to alcohol
43 Colorectal cancer
46 Other neoplasms

Leading causes 2006	Mean % change number of deaths 1990-2006	Mean % change all-age death rate 1990-2006	Mean % change age-standardised death rate 1990-2006
1 Ischaemic heart disease	**63.6%**	**27.5%**	6.6%
2 Cerebrovascular disease	**66.1%**	**29.5%**	5.7%
3 Tuberculosis	**-20.7%**	**-38.2%**	**-46.4%**
4 Diarrhoeal diseases	**-56.2%**	**-65.9%**	**-63.1%**
5 Diabetes	**98.0%**	**54.3%**	**29.7%**
6 Lower respiratory infections	**-37.9%**	**-51.6%**	**-38.1%**
7 COPD	**54.0%**	**20.0%**	-4.1%
8 Road injuries	5.7%	**-17.6%**	**-22.1%**
9 Neonatal preterm birth complications	**-21.7%**	**-39.0%**	**-29.3%**
10 Asthma	**14.7%**	-10.6%	**-24.7%**
11 Alzheimer's disease	**103.1%**	**58.3%**	**21.5%**
12 Chronic kidney disease	**64.1%**	**27.9%**	9.9%
13 Hypertensive heart disease	**31.1%**	2.1%	-13.5%
14 Neonatal encephalopathy	**-19.7%**	**-37.5%**	**-27.5%**
15 Peptic ulcer disease	**19.1%**	-7.2%	**-19.4%**
16 Congenital defects	-10.6%	-30.3%	-20.4%
17 Falls	**29.2%**	0.7%	-10.0%
18 Cirrhosis other	**40.0%**	9.1%	1.5%
19 Breast cancer	**62.7%**	**26.8%**	6.7%
20 Ileus & obstruction	**37.2%**	6.9%	9.5%
21 Protein-energy malnutrition	13.7%	-11.4%	-12.7%
22 Other cardiovascular diseases	**33.4%**	3.9%	-6.5%
23 Intestinal infectious diseases	**-29.3%**	**-44.9%**	**-38.8%**
24 Cirrhosis due to hepatitis B	**61.4%**	**25.8%**	5.2%
25 Stomach cancer	**34.0%**	4.4%	**-11.5%**
26 Lung cancer	**55.5%**	**21.2%**	0.6%
27 Cirrhosis due to alcohol	**68.9%**	**31.6%**	9.4%
28 Rheumatic heart disease	**-23.4%**	**-40.3%**	**-44.2%**
29 Drowning	-16.6%	**-35.0%**	**-27.6%**
30 Cirrhosis due to hepatitis C	**50.2%**	**17.0%**	-1.5%
31 Colorectal cancer			
33 Other neoplasms			
38 Neonatal sepsis			
40 Meningitis			
41 Measles			
43 Tetanus			
44 Malaria			

Leading causes 2016	Mean % change number of deaths 2006-2016	Mean % change all-age death rate 2006-2016	Mean % change age-standardised death rate 2006-2016
1 Ischaemic heart disease	**11.3%**	-0.5%	**-12.7%**
2 Cerebrovascular disease	**32.2%**	**18.2%**	0.5%
3 Tuberculosis	**-26.5%**	**-34.2%**	**-41.2%**
4 Diabetes	**54.2%**	**37.9%**	**16.5%**
5 COPD	**14.9%**	2.8%	**-11.9%**
6 Diarrhoeal diseases	-28.2%	**-35.8%**	**-31.7%**
7 Alzheimer's disease	**40.1%**	**25.3%**	**9.2%**
8 Road injuries	-4.9%	**-14.9%**	**-23.1%**
9 Lower respiratory infections	**-32.8%**	**-39.9%**	**-29.4%**
10 Chronic kidney disease	**16.1%**	3.9%	-11.8%
11 Asthma	-1.9%	**-12.3%**	**-23.6%**
12 Hypertensive heart disease	5.2%	-5.9%	**-16.0%**
13 Neonatal preterm birth complications	**-36.8%**	**-43.5%**	**-29.0%**
14 Peptic ulcer disease	-2.2%	**-12.5%**	**-21.5%**
15 Neonatal encephalopathy	**-39.5%**	**-45.9%**	**-32.0%**
16 Breast cancer	**19.1%**	6.5%	-9.7%
17 Falls	-0.4%	-11.0%	-19.2%
18 Cirrhosis other	4.3%	-6.7%	**-15.6%**
19 Ileus & obstruction	**12.3%**	0.4%	-1.8%
20 Protein-energy malnutrition	**12.7%**	0.8%	-1.7%
21 Congenital defects	**-33.6%**	**-40.7%**	**-28.1%**
22 Cirrhosis due to hepatitis B	**13.6%**	1.6%	**-11.9%**
23 Other cardiovascular diseases	5.2%	-5.9%	**-13.8%**
24 Lung cancer	**19.4%**	6.8%	**-9.6%**
25 Cirrhosis due to alcohol	**16.0%**	3.7%	**-11.4%**
26 Stomach cancer	**12.7%**	0.8%	**-12.1%**
27 Intestinal infectious diseases	**-8.6%**	**-18.2%**	**-12.8%**
28 Colorectal cancer	**23.9%**	**10.8%**	-3.9%
29 Cirrhosis due to hepatitis C	**11.6%**	-0.2%	**-14.2%**
30 Other neoplasms	**28.0%**	**14.5%**	3.9%
35 Rheumatic heart disease			
36 Drowning			
48 Malaria			
49 Neonatal sepsis			
52 Meningitis			
59 Measles			
76 Tetanus			

Legend:
Communicable, maternal, neonatal, and nutritional
Non-communicable
Injuries

SI Figure 3. Age-standardised rates of YLLs for males versus females in Indonesia and comparator countries, (A) 1990, (B) 2016

YLLs=years of life lost. HIV/AIDS=human immunodeficiency virus/acquired immunodeficiency syndrome.

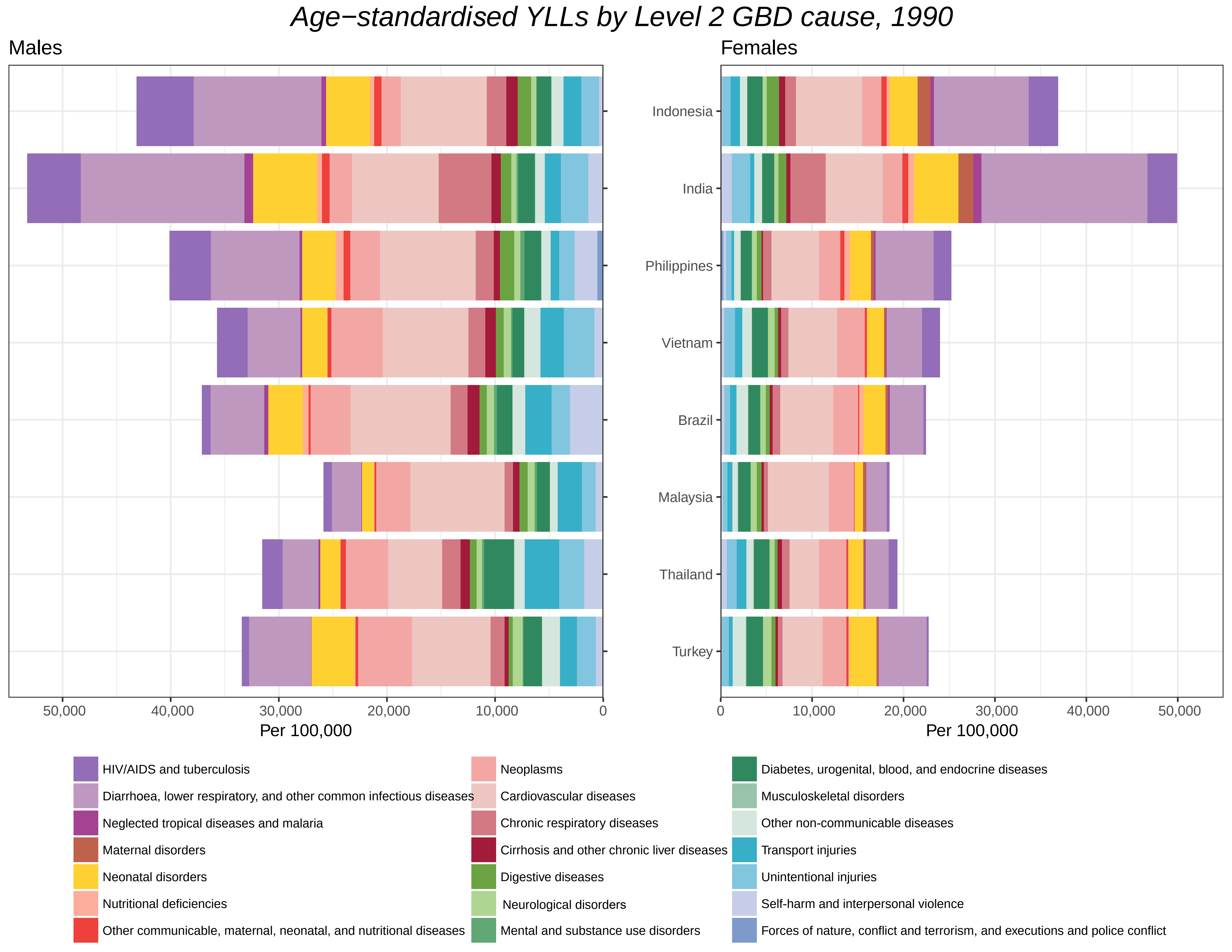

Age−standardised YLLs by Level 2 GBD cause, 1990
Males
Females
Indonesia
India
Philippines
Vietnam
Brazil
Malaysia
Thailand
Turkey
50,000
40,000
30,000
20,000
10,000
0
Per 100,000
0
10,000
20,000
30,000
40,000
50,000
Per 100,000
HIV/AIDS and tuberculosis
Diarrhoea, lower respiratory, and other common infectious diseases
Neglected tropical diseases and malaria
Maternal disorders
Neonatal disorders
Nutritional deficiencies
Other communicable, maternal, neonatal, and nutritional diseases
Neoplasms
Cardiovascular diseases
Chronic respiratory diseases
Cirrhosis and other chronic liver diseases
Digestive diseases
Neurological disorders
Mental and substance use disorders
Diabetes, urogenital, blood, and endocrine diseases
Musculoskeletal disorders
Other non-communicable diseases
Transport injuries
Unintentional injuries
Self-harm and interpersonal violence
Forces of nature, conflict and terrorism, and executions and police conflict

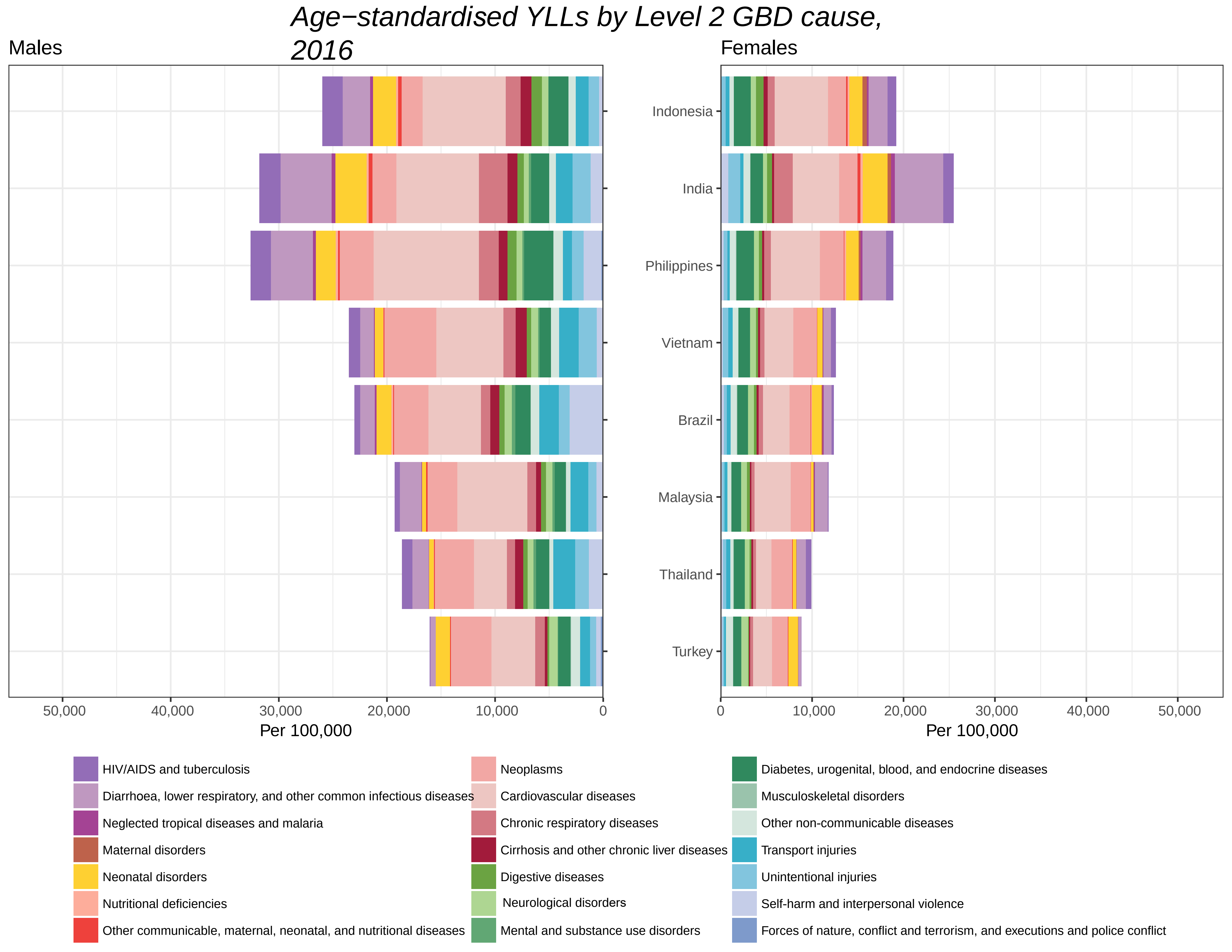
Age−standardised YLLs by Level 2 GBD cause, 2016
Males
Females
Indonesia
India
Philippines
Vietnam
Brazil
Malaysia
Thailand
Turkey
50,000
40,000
30,000
20,000
10,000
0
Per 100,000
HIV/AIDS and tuberculosis
Diarrhoea, lower respiratory, and other common infectious diseases
Neglected tropical diseases and malaria
Maternal disorders
Neonatal disorders
Nutritional deficiencies
Other communicable, maternal, neonatal, and nutritional diseases
Neoplasms
Cardiovascular diseases
Chronic respiratory diseases
Cirrhosis and other chronic liver diseases
Digestive diseases
Neurological disorders
Mental and substance use disorders
Diabetes, urogenital, blood, and endocrine diseases
Musculoskeletal disorders
Other non-communicable diseases
Transport injuries
Unintentional injuries
Self-harm and interpersonal violence
Forces of nature, conflict and terrorism, and executions and police conflict

SI Figure 4. Leading 30 Level 3 causes of YLLs in Indonesia for 1990, 2006, and 2016, with percent change in number of YLLs, and all-age and age-standardised YLL rates

Causes are ordered by total YLLs and are connected by arrows between time periods. Communicable, maternal, neonatal, and nutritional disorders are shown in red, non-communicable causes in blue, and injuries in green. For the time period 1990 to 2006 and for 2006 to 2015, three measures of change are shown: median percent change in the number of YLLs, median percent change in the all-age YLL rate, and median percent change in the age-standardised YLL rate. Median values across the 1,000 draws from the uncertainty distribution are shown. Both sexes combined. YLLs=years of life lost. COPD=chronic obstructive pulmonary disease. STDs=sexually transmitted diseases. HIV/AIDS=human immunodeficiency virus/acquired immunodeficiency syndrome.

Top 30 Level 3 causes of YLLs for 1990, 2006, and 2016, Indonesia, both sexes

Causes are connected by arrows between time periods. Communicable, maternal, neonatal, and nutritional disorders are shown in red, non-communicable causes in blue, and injuries in green. For the time period 1990 to 2016, three measures of change are shown: percent change in the number of YLLs, percent change in the all-age YLL rate, and percent change in the age-standardised YLL rate. Numbers in bold highlight statistically significant changes.

Leading causes 1990	Leading causes 2006	Mean % change number of YLLs 1990-2006	Mean % change all-age YLL rate 1990-2006	Mean % change age-standardised YLL rate 1990-2006	Leading causes 2016	Mean % change number of YLLs 2006-2016	Mean % change all-age YLL rate 2006-2016	Mean % change age-standardised YLL rate 2006-2016
1 Diarrhoeal diseases	1 Ischaemic heart disease	**56.6%**	**22.0%**	3.4%	1 Ischaemic heart disease	**9.8%**	-1.8%	**-15.3%**
2 Lower respiratory infections	2 Tuberculosis	**-27.1%**	**-43.2%**	**-48.3%**	2 Cerebrovascular disease	**29.3%**	**15.7%**	0.6%
3 Tuberculosis	3 Cerebrovascular disease	**54.5%**	**20.4%**	4.4%	3 Tuberculosis	**-30.3%**	**-37.6%**	**-41.9%**
4 Neonatal preterm birth complications	4 Lower respiratory infections	**-49.5%**	**-60.6%**	**-53.7%**	4 Diabetes	**54.3%**	**38.0%**	**16.3%**
5 Ischaemic heart disease	5 Neonatal preterm birth complications	**-21.7%**	**-39.0%**	**-29.3%**	5 Neonatal preterm birth complications	**-36.8%**	**-43.5%**	**-28.9%**
6 Neonatal encephalopathy	6 Diarrhoeal diseases	**-65.9%**	**-73.5%**	**-69.8%**	6 Road injuries	-6.5%	**-16.4%**	**-18.2%**
7 Measles	7 Neonatal encephalopathy	**-19.7%**	**-37.5%**	**-27.5%**	7 Lower respiratory infections	**-50.0%**	**-55.3%**	**-46.6%**
8 Cerebrovascular disease	8 Road injuries	-6.7%	**-27.3%**	**-25.4%**	8 Diarrhoeal diseases	**-48.6%**	**-54.0%**	**-46.9%**
9 Road injuries	9 Congenital defects	-11.3%	-30.9%	-20.9%	9 Neonatal encephalopathy	**-39.5%**	**-45.9%**	**-32.0%**
10 Congenital defects	10 Diabetes	**89.5%**	**47.7%**	**27.5%**	10 Congenital defects	**-34.3%**	**-41.3%**	**-28.3%**
11 Tetanus	11 Chronic kidney disease	**50.1%**	**17.0%**	6.5%	11 Chronic kidney disease	**14.3%**	2.2%	**-8.9%**
12 Intestinal infectious diseases	12 Intestinal infectious diseases	**-30.6%**	**-45.9%**	**-38.0%**	12 COPD	**14.6%**	2.5%	**-11.1%**
13 Drowning	13 Asthma	3.4%	**-19.4%**	**-26.6%**	13 Asthma	-5.7%	**-15.6%**	**-24.7%**
14 Asthma	14 COPD	**47.0%**	**14.5%**	-4.1%	14 Intestinal infectious diseases	**-11.4%**	**-20.7%**	**-14.1%**
15 Diabetes	15 Drowning	-24.3%	**-41.0%**	**-34.0%**	15 Hypertensive heart disease	2.7%	-8.2%	**-20.1%**
16 Neonatal sepsis	16 Hypertensive heart disease	**23.7%**	-3.6%	-17.5%	16 Breast cancer	**18.4%**	5.9%	-10.5%
17 Other neonatal	17 Neonatal sepsis	-18.0%	**-36.1%**	-26.1%	17 Alzheimer's disease	**37.6%**	**23.1%**	**7.6%**
18 Meningitis	18 Other neonatal disorders	-16.1%	**-34.6%**	**-24.4%**	18 Cirrhosis other	-2.0%	**-12.4%**	**-17.9%**
19 Chronic kidney disease	19 Ileus & obstruction	6.0%	-17.4%	-7.6%	19 Ileus & obstruction	-9.9%	**-19.4%**	**-13.4%**
20 STDs	20 Cirrhosis other	**21.4%**	-5.4%	-7.1%	20 Peptic ulcer disease	**-7.6%**	**-17.4%**	**-25.2%**
21 Rheumatic heart disease	21 Peptic ulcer disease	4.6%	**-18.4%**	**-25.5%**	21 Falls	-7.6%	**-17.3%**	**-21.0%**
22 COPD	22 Measles	**-81.1%**	**-85.3%**	**-83.3%**	22 Drowning	**-29.3%**	**-36.8%**	**-31.8%**
23 Malaria	23 Falls	11.9%	-12.8%	**-15.8%**	23 Cirrhosis due to hepatitis B	**10.7%**	-1.0%	**-13.6%**
24 Ileus & obstruction	24 Meningitis	-28.4%	**-44.2%**	**-37.3%**	24 Neonatal sepsis	**-31.9%**	**-39.0%**	**-23.4%**
25 Peptic ulcer disease	25 Breast cancer	**60.6%**	**25.1%**	3.7%	25 Self-harm	0.5%	**-10.1%**	**-10.6%**
26 Hypertensive heart disease	26 Self-harm	**31.3%**	2.3%	-3.8%	26 HIV/AIDS	**132.5%**	**107.9%**	**103.8%**
27 Cirrhosis other	27 Alzheimer's disease	**90.2%**	**48.2%**	**19.1%**	27 Cirrhosis due to alcohol	**13.9%**	1.9%	**-13.0%**
28 Falls	28 Cirrhosis due to hepatitis B	**58.8%**	**23.8%**	2.8%	28 Other neoplasms	**18.0%**	5.5%	-0.2%
29 Protein-energy malnutrition	29 Rheumatic heart disease	**-33.2%**	**-48.0%**	**-49.0%**	29 Dengue	31.1%	17.2%	30.3%
30 Whooping cough	30 Other cardiovascular	**14.2%**	-11.0%	**-14.2%**	30 Other cardiovascular diseases	-3.9%	**-14.1%**	**-19.2%**
34 Other cardiovascular diseases	31 Whooping cough				31 Other neonatal disorders			
36 Self-harm	33 Cirrhosis due to alcohol				34 Measles			
37 Breast cancer	34 Protein-energy malnutrition				38 Meningitis			
38 Cirrhosis due to hepatitis B	35 Other neoplasms				40 Rheumatic heart disease			
41 Alzheimer's disease	37 Tetanus				41 Protein-energy malnutrition			
44 Dengue	38 STDs				47 Malaria			
48 Other neoplasms	42 Dengue				48 Whooping cough			
49 Cirrhosis due to alcohol	43 Malaria				56 STDs			
137 HIV/AIDS	60 HIV/AIDS				69 Tetanus			

Legend:
Communicable, maternal, neonatal, and nutritional
Non-communicable
Injuries

SI Figure 5. Leading 30 Level 3 causes of YLDs in Indonesia for 1990, 2006, and 2016, with percent change in number of YLDs, and all-age and age-standardised YLD rates

Causes are ordered by total YLDs and are connected by arrows between time periods. Communicable, maternal, neonatal, and nutritional disorders are shown in red, non-communicable causes in blue, and injuries in green. For the time period 1990 to 2006 and for 2006 to 2016, three measures of change are shown: median percent change in the number of YLDs, median percent change in the all-age YLD rate, and median percent change in the age-standardised YLD rate. Median values across the 1,000 draws from the uncertainty distribution are shown. Both sexes combined. YLDs=years lived with disability. COPD=chronic obstructive pulmonary disease. STDs=sexually transmitted diseases.

Top 30 Level 3 causes of YLDs for 1990, 2006, and 2016, Indonesia, both sexes

Causes are connected by arrows between time periods. Communicable, maternal, neonatal, and nutritional disorders are shown in red, non-communicable causes in blue, and injuries in green. For the time period 1990 to 2016, three measures of change are shown: percent change in the number of YLDs, percent change in the all-age YLD rate, and percent change in the age-standardised YLD rate. Numbers in bold highlight statistically significant changes.

Leading causes 1990	Leading causes 2006	Mean % change number of YLDs 1990-2006	Mean % change all-age YLD rate 1990-2006	Mean % change age-standardised YLD rate 1990-2006	Leading causes 2016	Mean % change number of YLDs 2006-2016	Mean % change all-age YLD rate 2006-2016	Mean % change age-standardised YLD rate 2006-2016
1 Low back & neck pain	1 Low back & neck pain	**46.3%**	**14.0%**	-1.4%	1 Low back & neck pain	**22.1%**	**9.2%**	-1.7%
2 Skin diseases	2 Sense organ diseases	**40.1%**	**9.2%**	**-3.2%**	2 Sense organ diseases	**19.8%**	**7.2%**	**-3.6%**
3 Sense organ diseases	3 Skin diseases	**24.4%**	**-3.0%**	**1.7%**	3 Skin diseases	**11.5%**	-0.3%	**2.4%**
4 Migraine	4 Migraine	**38.5%**	**7.9%**	-0.5%	4 Migraine	**15.3%**	**3.1%**	-0.8%
5 Iron-deficiency anaemia	5 Iron-deficiency anaemia	**17.5%**	**-8.5%**	**-6.8%**	5 Iron-deficiency anaemia	**5.7%**	**-5.4%**	**-3.8%**
6 Depressive disorders	6 Depressive disorders	**37.4%**	**7.1%**	**-2.3%**	6 Diabetes	**56.2%**	**39.7%**	**20.9%**
7 Anxiety disorders	7 Other musculoskeletal disorders	**45.1%**	**13.1%**	-0.3%	7 Depressive disorders	**19.8%**	**7.1%**	-0.1%
8 Other musculoskeletal disorders	8 Anxiety disorders	**35.5%**	**5.6%**	0.3%	8 Other musculoskeletal disorders	**19.8%**	**7.2%**	-1.5%
9 Asthma	9 Diabetes	**65.7%**	**29.1%**	**9.9%**	9 Anxiety disorders	**16.1%**	3.9%	0.0%
10 Diabetes	10 Asthma	**24.4%**	**-3.1%**	-0.8%	10 COPD	**38.6%**	**23.9%**	**6.9%**
11 Diarrhoeal diseases	11 Oral disorders	**45.3%**	**13.2%**	0.1%	11 Oral disorders	**23.5%**	**10.4%**	-0.2%
12 Oral disorders	12 COPD	**72.0%**	**34.1%**	**13.8%**	12 Asthma	**17.5%**	**5.1%**	**3.2%**
13 COPD	13 Schizophrenia	**51.4%**	**18.0%**	2.3%	13 Schizophrenia	**20.7%**	**8.0%**	1.6%
14 Tuberculosis	14 Diarrhoeal diseases	**-10.9%**	**-30.5%**	**-28.5%**	14 Osteoarthritis	**39.3%**	**24.6%**	**4.3%**
15 Intestinal nematodes	15 Epilepsy	29.6%	1.0%	3.9%	15 Stroke	**40.4%**	**25.6%**	**8.5%**
16 Schizophrenia	16 Osteoarthritis	**64.0%**	**27.8%**	**5.5%**	16 Epilepsy	16.4%	4.1%	4.9%
17 Epilepsy	17 Stroke	**64.6%**	**28.3%**	**9.5%**	17 Diarrhoeal diseases	**3.9%**	**-7.1%**	**-5.9%**
18 Autistic spectrum disorders	18 Other mental & substance use	**46.4%**	**14.1%**	0.2%	18 Other mental & substance use	**18.9%**	**6.4%**	-0.1%
19 Lymphatic filariasis	19 Autistic spectrum disorders	**26.8%**	-1.2%	0.4%	19 Autistic spectrum disorders	**11.0%**	-0.8%	0.4%
20 Conduct disorder	20 Lymphatic filariasis	**19.9%**	**-6.5%**	**-16.1%**	20 Drug use disorders	**16.8%**	**4.4%**	**6.1%**
21 Other mental & substance use	21 Drug use disorders	**40.6%**	**9.6%**	**3.8%**	21 Gynaecological diseases	**22.7%**	**9.7%**	**3.3%**
22 Osteoarthritis	22 Neonatal preterm birth complications	**39.8%**	**8.9%**	**12.9%**	22 Falls	**38.7%**	**24.1%**	**11.9%**
23 Drug use disorders	23 Gynaecological diseases	**40.2%**	**9.2%**	**-5.9%**	23 Bipolar disorder	**16.2%**	**3.9%**	-0.1%
24 Neonatal preterm birth complications	24 Bipolar disorder	**38.5%**	**7.9%**	0.1%	24 Neonatal preterm birth complications	9.2%	-2.4%	0.7%
25 Gynaecological diseases	25 Conduct disorder	**7.2%**	**-16.5%**	0.3%	25 Road injuries	**65.4%**	**47.9%**	**32.4%**
26 Congenital defects	26 Tuberculosis	**-16.1%**	**-34.6%**	**-37.0%**	26 Ischaemic heart disease	**33.2%**	**19.2%**	**2.1%**
27 Bipolar disorder	27 Upper respiratory infections	**30.9%**	2.0%	**3.9%**	27 Conduct disorder	**4.1%**	**-6.9%**	0.4%
28 Stroke	28 Congenital defects	**21.8%**	**-5.1%**	**-2.9%**	28 Upper respiratory infections	**9.0%**	**-2.5%**	0.1%
29 Upper respiratory infections	29 Falls	**44.7%**	**12.7%**	1.4%	29 Congenital defects	**7.0%**	**-4.3%**	**-2.6%**
30 Falls	30 Ischaemic heart disease	**70.4%**	**32.8%**	**10.8%**	30 Tuberculosis	2.5%	**-8.3%**	**-16.4%**
35 Ischaemic heart disease	33 Road injuries				38 Lymphatic filariasis			
37 Road injuries	46 Intestinal nematodes				47 Intestinal nematodes			

Legend:
Communicable, maternal, neonatal, and nutritional
Non-communicable
Injuries

SI Figure 6. Leading 30 Level 3 risk factors for YLLs in Indonesia for 1990, 2006, and 2016, with percent change in number of YLLs, and all-age and age-standardised YLL rates

Risks are ordered by total YLLs and are connected by arrows between time periods. Environmental and occupational risks are shown in blue, metabolic risks are shown in yellow, and behavioural risks are shown in purple. For the time period 1990 to 2006 and for 2006 to 2016, three measures of change are shown: median percent change in the number of YLLs, median percent change in the all-age YLL rate, and median percent change in the age-standardised YLL rate. Median values across the 1,000 draws from the uncertainty distribution are shown. Both sexes combined. YLLs=years of life lost. Low PUFA=low polyunsaturated fatty acids.

Leading 30 GBD Level 3 risks of YLLs for both sexes combined for 1990 and 2006, Indonesia

Leading causes 1990	Leading causes 2006	Mean % change number of YLLs 1990-2006	Mean % change all-age YLL rate 1990-2006	Mean % change age-standardised YLL rate 1990-2006	Leading causes 2016	Mean % change number of YLLs 2006-2016	Mean % change all-age YLL rate 2006-2016	Mean % change age-standardised YLL rate 2006-2016
1 Child growth failure	1 High blood pressure	**62.4%**	**26.5%**	5.6%	1 High blood pressure	**19.6%**	**7.0%**	**-8.8%**
2 Low birth weight & short gestation	2 Low birth weight & short gest.	**-28.4%**	**-44.2%**	**-35.4%**	2 High fasting plasma glucose	**42.4%**	**27.3%**	**8.7%**
3 Unsafe sanitation	3 Smoking	**48.8%**	**15.9%**	-3.8%	3 Smoking	**18.3%**	5.8%	**-9.1%**
4 Unsafe water	4 Child growth failure	**-65.0%**	**-72.7%**	**-69.4%**	4 High body-mass index	**63.7%**	**46.4%**	**25.4%**
5 Household air pollution	5 High fasting plasma glucose	**72.3%**	**34.3%**	**14.9%**	5 Low birth weight & short gest.	**-38.7%**	**-45.2%**	**-31.0%**
6 High blood pressure	6 Household air pollution	**-32.5%**	**-47.4%**	**-42.2%**	6 Low whole grains	**23.1%**	**10.1%**	**-5.2%**
7 Smoking	7 Low whole grains	**60.8%**	**25.3%**	5.1%	7 High total cholesterol	**12.4%**	0.5%	**-14.1%**
8 High fasting plasma glucose	8 High total cholesterol	**57.2%**	**22.5%**	2.7%	8 Low fruit	**8.9%**	-2.6%	**-16.4%**
9 Handwashing	9 High body-mass index	**170.9%**	**111.1%**	**74.1%**	9 Low nuts and seeds	**13.2%**	1.3%	**-13.1%**
10 Suboptimal breastfeeding	10 Low fruit	**42.3%**	**10.9%**	-7.7%	10 Ambient particulate matter	0.9%	**-9.7%**	**-16.4%**
11 Ambient particulate matter	11 Ambient particulate matter	4.2%	**-18.8%**	**-15.1%**	11 Low vegetables	**7.7%**	-3.7%	**-16.8%**
12 Low whole grains	12 Low vegetables	**47.8%**	**15.2%**	-3.2%	12 Impaired kidney function	**19.3%**	**6.7%**	**-7.4%**
13 High total cholesterol	13 Low nuts and seeds	**59.9%**	**24.6%**	4.7%	13 Household air pollution	**-47.5%**	**-53.1%**	**-55.4%**
14 Secondhand smoke	14 Unsafe water	**-66.0%**	**-73.5%**	**-69.8%**	14 Child growth failure	**-61.8%**	**-65.9%**	**-58.8%**
15 Low fruit	15 Impaired kidney function	**56.7%**	**22.1%**	7.0%	15 Occupational injury	-0.6%	**-11.1%**	**-13.0%**
16 Low vegetables	16 Occupational injury	**21.0%**	-5.7%	**-14.4%**	16 Low omega-3	**6.3%**	-5.0%	**-18.4%**
17 Occupational injury	17 Low omega-3	**53.4%**	**19.6%**	0.5%	17 Secondhand smoke	**-16.3%**	**-25.1%**	**-26.1%**
18 Low nuts and seeds	18 Unsafe sanitation	**-74.9%**	**-80.4%**	**-77.7%**	18 Low fibre	3.2%	**-7.7%**	**-20.3%**
19 Impaired kidney function	19 Secondhand smoke	**-26.8%**	**-43.0%**	**-32.7%**	19 Unsafe water	**-48.2%**	**-53.7%**	**-46.5%**
20 High body-mass index	20 Low fibre	**50.4%**	**17.2%**	-1.9%	20 Low physical activity	17.6%	5.2%	-9.3%
21 Low omega-3	21 Low physical activity	**61.2%**	25.6%	4.5%	21 Low PUFA	**11.9%**	0.1%	**-12.9%**
22 Vitamin A deficiency	22 Handwashing	**-65.9%**	**-73.5%**	**-70.1%**	22 Alcohol use	11.7%	-0.1%	**-13.3%**
23 Low fibre	23 Suboptimal breastfeeding	**-65.7%**	**-73.3%**	**-69.5%**	23 Low legumes	**19.2%**	6.6%	**-8.2%**
24 Low physical activity	24 Alcohol use	**47.7%**	**15.1%**	-2.0%	24 Unsafe sex	**46.1%**	**30.6%**	**11.1%**
25 Alcohol use	25 Low PUFA	**47.8%**	**15.2%**	-2.9%	25 Occupational carcinogens	**35.9%**	**21.5%**	2.0%
26 Low PUFA	26 Low legumes	**158.8%**	**101.7%**	**66.2%**	26 High sodium	-5.0%	**-15.0%**	**-26.6%**
27 High sodium	27 High sodium	49.1%	16.2%	-3.1%	27 Unsafe sanitation	**-62.1%**	**-66.1%**	**-60.9%**
28 Zinc deficiency	28 Occupational carcinogens	**83.6%**	**43.1%**	**21.2%**	28 Handwashing	**-49.0%**	**-54.4%**	**-47.0%**
29 Occupational carcinogens	29 Unsafe sex	**103.2%**	**58.4%**	**24.0%**	29 Drug use	**40.0%**	**25.2%**	**13.0%**
30 Low legumes	30 Lead	**68.4%**	**31.2%**	**15.6%**	30 Suboptimal breastfeeding	**-64.8%**	**-68.5%**	**-61.2%**
31 Unsafe sex	31 Drug use				31 Lead			
32 Lead	33 Vitamin A deficiency				35 Vitamin A deficiency			
34 Drug use	36 Zinc deficiency				40 Zinc deficiency			

Legend:
Environmental/occupational risks
Metabolic risks
Behavioral risks

SI Figure 7. Ratio of observed to expected age-standardised YLLs, Indonesia and comparator countries, 1990 and 2016, with uncertainty intervals

Ratio of observed to expected age-standardised YLLs, Indonesia and comparator countries, 1990 and 2016

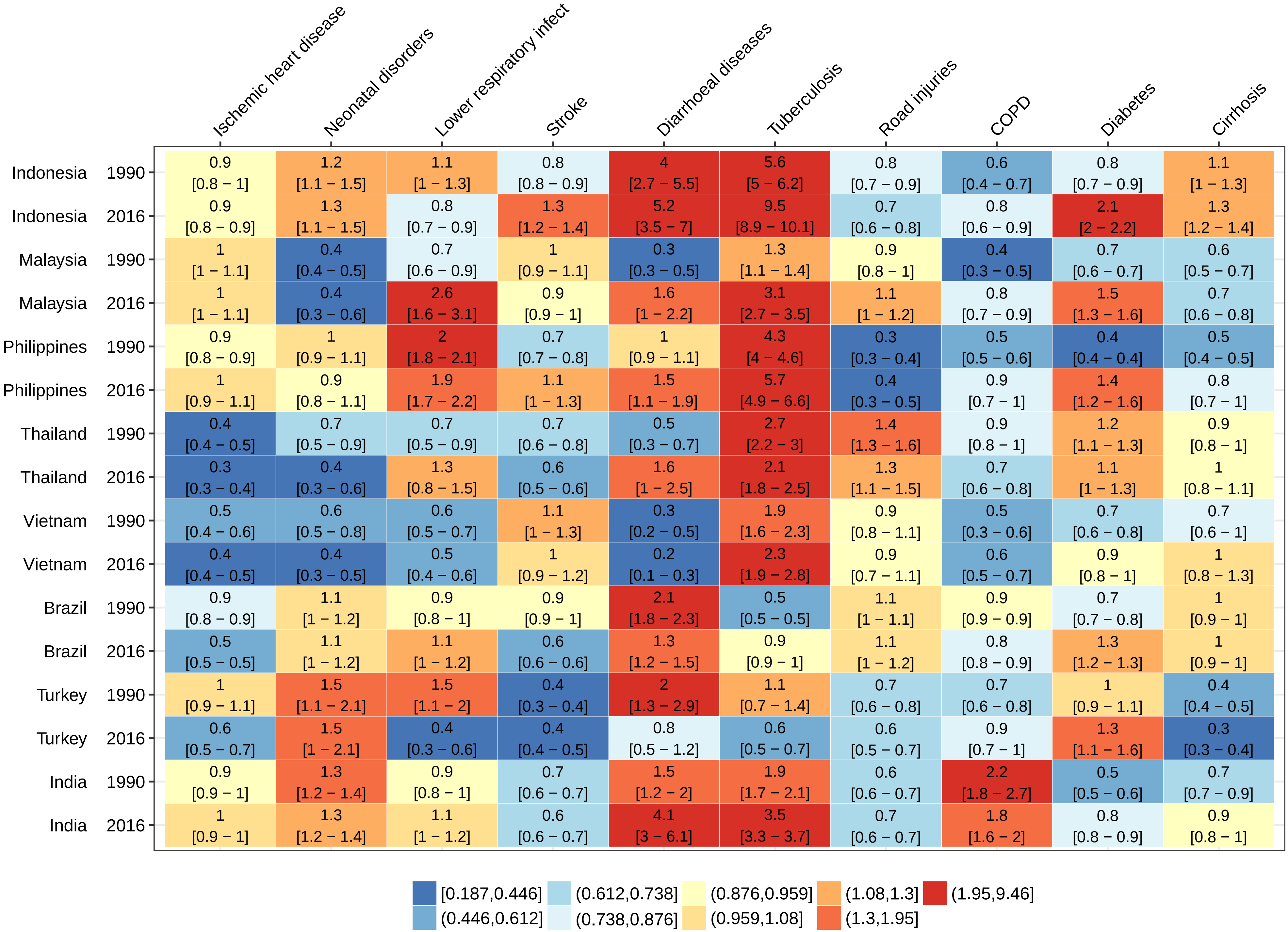

Supplement: Supplementary appendix [file mmc1.pdf]
